# A Cell-Permeant Mimetic of NMN Activates SARM1 to Produce Cyclic ADP-Ribose and Induce Non-apoptotic Cell Death

**DOI:** 10.1016/j.isci.2019.05.001

**Published:** 2019-05-04

**Authors:** Zhi Ying Zhao, Xu Jie Xie, Wan Hua Li, Jun Liu, Zhe Chen, Ben Zhang, Ting Li, Song Lu Li, Jun Gang Lu, Liangren Zhang, Li-he Zhang, Zhengshuang Xu, Hon Cheung Lee, Yong Juan Zhao

**Affiliations:** 1 State Key Laboratory of Chemical Oncogenomics, Key Laboratory of Chemical Genomics, School of Chemical Biology and Biotechnology, Peking University Shenzhen Graduate School, Shenzhen 518055, China; 2 State Key Laboratory of Natural and Biomimetic Drugs, School of Pharmaceutical Sciences, Peking University, Beijing 100191, China; 3 Agilent Technologies (China) Co.,Ltd, Guangzhou 510613, China

**Keywords:** Biochemistry, Enzymology, Biochemical Mechanism

## Abstract

SARM1, an NAD-utilizing enzyme, regulates axonal degeneration. We show that CZ-48, a cell-permeant mimetic of NMN, activated SARM1 *in vitro* and *in cellulo* to cyclize NAD and produce a Ca^2+^ messenger, cADPR, with similar efficiency as NMN. Knockout of NMN-adenylyltransferase elevated cellular NMN and activated SARM1 to produce cADPR, confirming NMN was its endogenous activator. Determinants for the activating effects and cell permeability of CZ-48 were identified. CZ-48 activated SARM1 via a conformational change of the auto-inhibitory domain and dimerization of its catalytic domain. SARM1 catalysis was similar to CD38, despite having no sequence similarity. Both catalyzed similar set of reactions, but SARM1 had much higher NAD-cyclizing activity, making it more efficient in elevating cADPR. CZ-48 acted selectively, activating SARM1 but inhibiting CD38. In SARM1-overexpressing cells, CZ-48 elevated cADPR, depleted NAD and ATP, and induced non-apoptotic death. CZ-48 is a specific modulator of SARM1 functions in cells.

## Highlights

• CZ-48, a cell-permeant mimetic of NMN, activates SARM1 but inhibits CD38 enzymatically
• SARM1 catalysis is similar to CD38, but with higher cyclase activity
• Activation by CZ-48 or NMN elicits conformational changes in SARM1
• Activation of SARM1 causes cADPR production, NAD depletion, and non-apoptotic cell death

## Graphical Abstract

**Figure:**
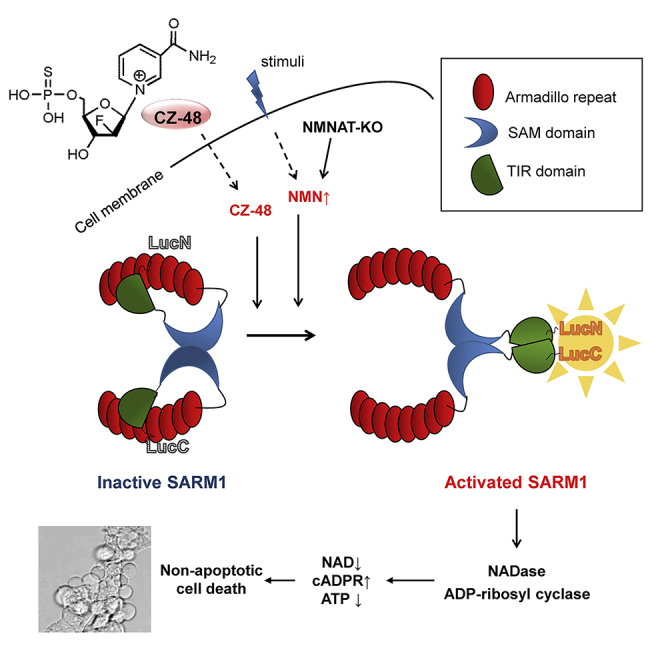

## Introduction

Sterile alpha and Toll/interleukin-1 receptor motif-containing 1 (SARM1) is an adaptor protein in the Toll-like receptor pathway (Carty et al., 2006). It plays an important role in mediating axonal degeneration, which is observed in many neurological disorders such as peripheral neuropathy, traumatic brain injury, and neurodegenerative diseases (Gerdts et al., 2016). After injury, nicotinamide mononucleotide (NMN) accumulates (Di Stefano et al., 2015) and intracellular Ca^2+^ rises in the injured axons (Loreto et al., 2015), followed by NAD depletion and axonal fragmentation. SARM1 is required in this process (Gerdts et al., 2015) as SARM1-knockout mice show neuroprotective effects after injury both in mice and *Drosophila* (Osterloh et al., 2012).

Surprisingly, recent studies (Essuman et al., 2017, Essuman et al., 2018) suggest that SARM1 is actually an enzyme with activities related to CD38 (Howard et al., 1993), a completely different protein mainly responsible for cyclizing NAD to cyclic ADP-ribose (cADPR) (Clapper et al., 1987, Lee et al., 1994). It is a novel cyclic nucleotide and a second messenger for mediating the mobilization of the endoplasmic Ca^2+^ stores by sensitizing the Ca^2+^-induced Ca^2+^ release activity of the ryanodine receptors (Galione et al., 1991, Lee, 1993). Ablation of the CD38 gene in mouse results in depletion of cADPR contents in many tissues (Partida-Sanchez et al., 2001) and leads to multiple physiological defects in insulin secretion, neutrophil chemotaxis, and oxytocin secretion (see review Malavasi et al., 2008). Interestingly, the cADPR contents in the brain of the CD38-knockout mice remain substantial (Partida-Sanchez et al., 2001), indicating the existence of an unknown cADPR-synthesizing enzyme. In this study, we identify SARM1 as such an enzyme.

CD38 and SARM1 have no sequence similarity, a large difference in size, distinct subcellular localizations, and yet both are NAD-utilizing enzymes. In lymphocytes, CD38 is expressed on the cell surface as a type II transmembrane protein (Jackson and Bell, 1990). It is also expressed intracellularly in the endoplasmic reticulum in an opposite orientation (type III), with the catalytic carboxyl domain facing the cytosol (Liu et al., 2017, Zhao et al., 2012). SARM1, on the other hand, is localized to the mitochondria (Panneerselvam et al., 2012), with its major portion facing the cytosol (Gerdts et al., 2013). The catalytic mechanism of CD38 has been well elucidated. We show by crystallography that NAD enters the active site and forms an intermediate with the catalytic residue, Glu226, at the C1 of the ribose, releasing the nicotinamide ring. Subsequent attack and linkage of C1 with the N1 of the adenine results in cyclization and produces cADPR (Lee, 2006, Liu et al., 2005, Liu et al., 2008a, 2008b). The intriguing question of whether SARM1 and CD38, two entirely different proteins, actually use a similar catalytic mechanism for producing cADPR is addressed in this study.

As CD38 regulates many physiological functions, great efforts have been focused in developing pharmacological reagents to manipulate its enzymatic activities (Becherer et al., 2015, Haffner et al., 2015, Kwong et al., 2012). We have synthesized a series of mimetics of NMN that form covalent intermediates with Glu226 of CD38 and inhibit its enzymatic activities (Kwong et al., 2012). During the studies, we unexpectedly observed that one of these inhibitors, sulfo-ara-F-NMN (CZ-48), could effectively elevate cellular cADPR contents in cells not expressing CD38. We document here that the enzyme activated by CZ-48 is SARM1. Endogenous NMN itself can also activate SARM1, pointing to its regulation by the NAD metabolic pathway. We also characterize the enzymatic activities of SARM1 and show that they are similar to CD38. Its activation by NMN and CZ-48 is determined to involve a conformational relieve of its auto-inhibitory domain. That CZ-48 is cell permeant and effective in activating SARM1 in cells makes it a valuable tool for manipulating its enzymatic activity and investigating its functions.

## Results

### An Inhibitor of CD38, CZ-48 Induces Intracellular cADPR Production

**Figure 1:**
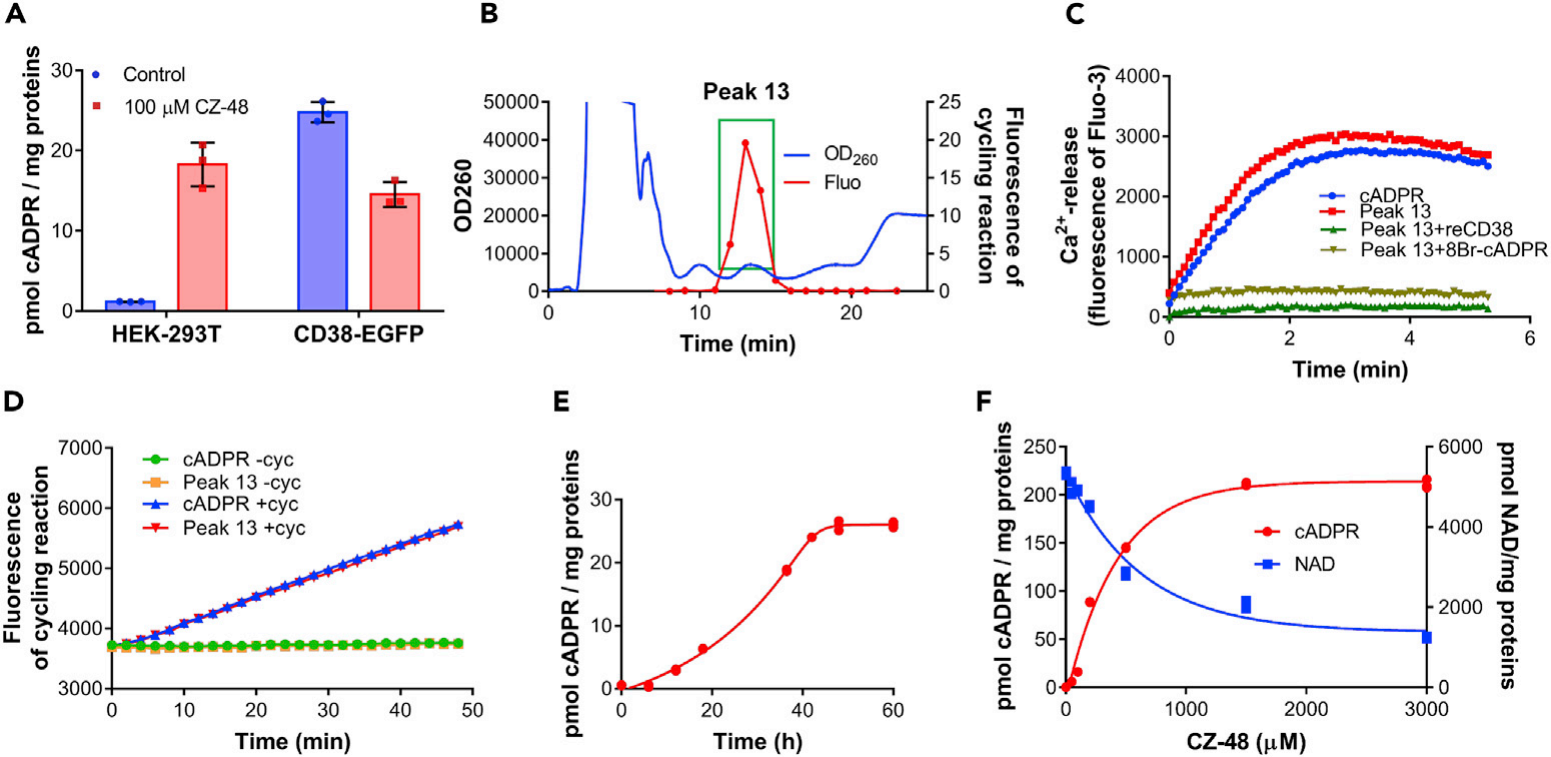
An Inhibitor of CD38, CZ-48 Induces Intracellular cADPR Production (A) Wild-type and CD38-EGFP-overexpressing HEK-293T cells were treated with 100 μM CZ-48 for 24 h, and cADPR contents were analyzed by cycling assay. (B) The target compound was separated by HPLC. HEK-293T cells were treated with 100 μM CZ-48 for 72 h, and the nucleotides were extracted and fractionated by HPLC with an AG MP-1 column (blue line, left y axis). Fractions 4, 5, and 6 (Peak 13, green box) showed positive signals in the cycling assay (red line, right y axis). (C) Peak 13 released Ca^2+^ from sea urchin homogenate similar to 0.5 μM cADPR, was blocked by 500 μM 8Br-cADPR pre-treatment of the homogenate, and was destroyed by 10 μg/mL reCD38 pre-treatment of the compound. (D) Peak 13 produced the fluorescence signals similar to 0.5 μM cADPR in cycling assay. (E) The time course of cADPR production in the CZ-48-treated HEK-293T cells. HEK-293T cells were treated with 100 μM CZ-48 for different time periods, and cADPR contents were analyzed by cycling assay. (F) Dose-response curves of CZ-48 in intracellular cADPR production and NAD consumption. HEK-293T cells were treated with different doses of CZ-48 for 24 h, and the amounts of cADPR and NAD were analyzed. All the above-mentioned experiments were repeated at least three times (means ± SDs; n = 3).

We have previously designed and synthesized a series of inhibitors of CD38 using arabinosyl-2′-fluoro-2′-deoxynicotinamide mononucleotide (ara-F-NMN or CZ-17, structure in Figure 3A) (Kwong et al., 2012, Sauve et al., 2000) as a template. They form covalent linkages with the catalytic residue, Glu226, of purified recombinant CD38 and can effectively inhibit its enzymatic activities in the submicromolar range. An example is CZ-48 (structure in Figure 3A), which is cell permeant and can inhibit EGFP-tagged CD38 (CD38-EGFP) stably expressed in HEK-293T cells (Zhao et al., 2011), causing decrease in intracellular cADPR levels (Figure 1A, right two bars).

Surprisingly, when the control HEK-293T cells, without detectable CD38, were treated with CZ-48, a large elevation of cellular cADPR was observed (Figure 1A, left two bars), reaching similar levels as the CD38-expressing cells. To ensure that the elevated signals detected by the fluorescence cycling assay for cADPR (Graeff and Lee, 2002) were indeed cADPR, the nucleotides of the treated cells were extracted and separated by high-performance liquid chromatography (HPLC) (Figure 1B, blue line). Fractions were collected and analyzed for cADPR (Figure 1B, red line). As shown in Figure 1B, the peak of the positive fractions showed a retention time (13 min) typical for the cADPR standard. Mass spectrometry confirmed that the m/z value of Peak 13 (combination of fractions 4, 5, and 6) was 542.02, same as that of cADPR (Figure S1A).

Furthermore, Peak 13 could release Ca^2+^ from the sea urchin homogenates (Figure 1C, red), the first and classical bioassay for cADPR (Lee et al., 1989), with activity equal to that of 0.5 μM cADPR standard (Figure 1C, blue; same concentration was determined in cycling assay, Figure 1D). The Ca^2+^-releasing activity of Peak 13 could be blocked with 8Br-cADPR (Figure 1C, pale green), a specific antagonist of cADPR (Walseth and Lee, 1993), and could be eliminated by pre-treatment with recombinant CD38 (reCD38), which is the specific hydrolase of cADPR (Figure 1C, green). Taken together, the results firmly established that the elevated cellular signals effectively activated by CZ-48 were actually cADPR.

The time course of the CZ-48-induced cADPR production in HEK-293T cells is shown in Figure 1E. The cellular cADPR contents progressively accumulated and reached a plateau in about 40 h after treatment with 100 μM CZ-48. The stimulatory effect of CZ-48 on cellular cADPR production was concentration dependent and saturable as shown in Figure 1F (red, left y axis). Corresponding to the elevation of cADPR, the cellular NAD levels dropped (Figure 1F, blue, right y axis), suggesting that cADPR was derived from NAD.

### The Effect of CZ-48 Was Not Mediated by CD38 or BST-1, but by SARM1

**Figure 2:**
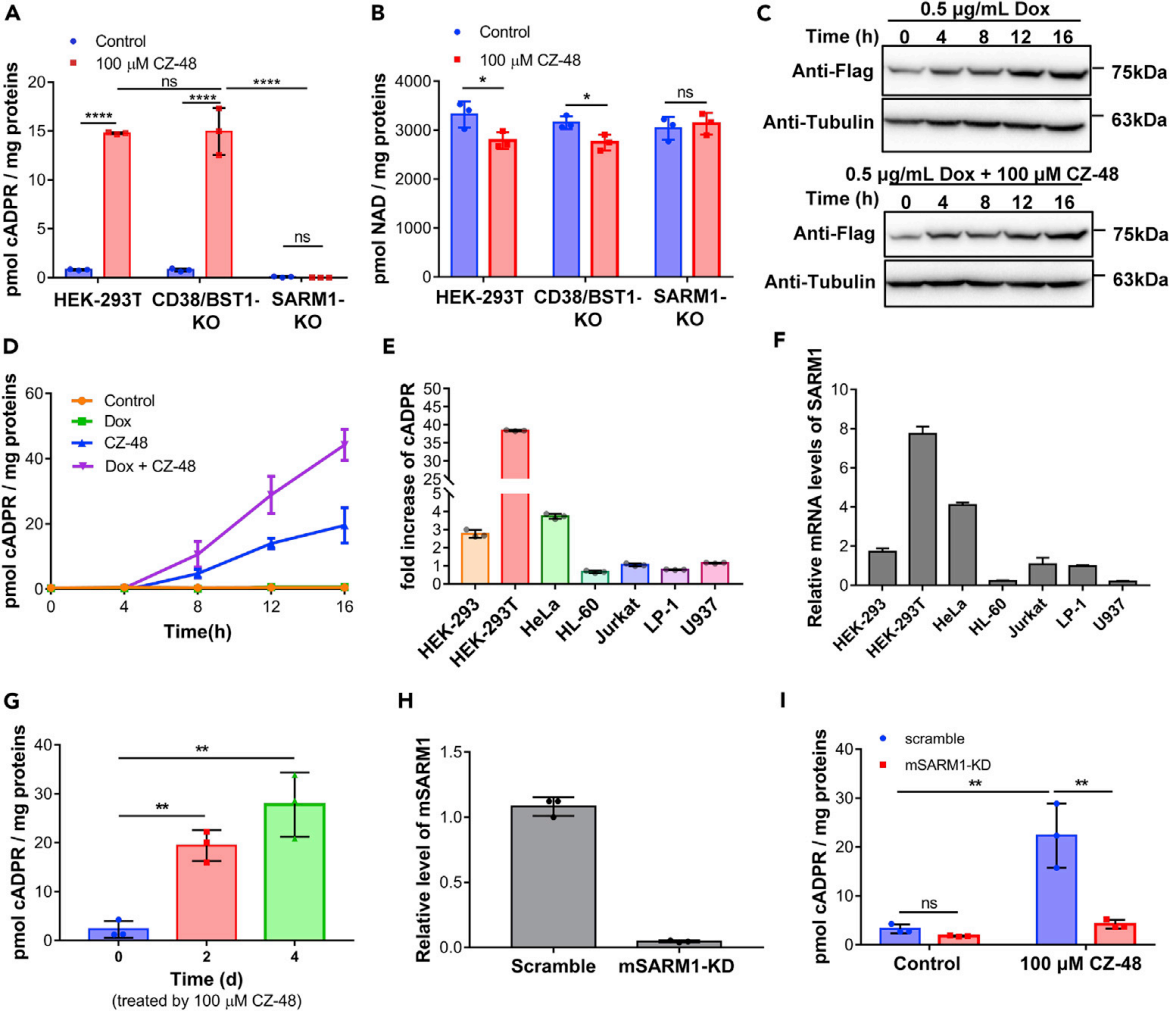
The Effect of CZ-48 Was Not Mediated by CD38 or BST-1, but by SARM1 (A and B) Wild-type, CD38/BST-1 double KO, and SARM1-KO HEK-293T cells were treated with 100 μM CZ-48 for 24 h, and intracellular cADPR (A) and NAD (B) contents were measured. (C and D) HEK-293 cells carrying an inducible expression cassette of FLAG-tagged SARM1 were treated with 100 μM CZ-48, 0.5 μg/mL Dox, or both for different time periods. The expression levels of SARM1-FLAG were analyzed by western blots (C), and the cADPR contents were analyzed by cycling assay (D). (E) The cellular cADPR levels were measured in different cell lines after treatment of 100 μM CZ-48 for 24 h, and the fold changes were presented. (F) The mRNA levels of SARM1 in the cell lines were quantified by qRT-PCR. (G) The primary culture of mouse sensory neurons was treated with 100 μM CZ-48 for 2 or 4 days, and the cADPR levels were measured. (H) SARM1 was knocked down in the mouse neurons, assayed by qRT-PCR. (I) SARM1-knockdown neurons, together with the scramble short hairpin RNA-infected cells as controls, were treated with 100 μM CZ-48 for 48 h, and the cADPR levels were measured. All the above experiments were repeated at least three times (means ± SDs; n = 3; Student's t test, *p < 0.05, **p < 0.01, ****p < 0.0001).

To determine the target of CZ-48, we used the TALEN (Bogdanove and Voytas, 2011) and CRISPR techniques (Ran et al., 2013) to delete CD38 and BST-1 in HEK-293T cells, the only two known mammalian enzymes that can cyclize NAD to cADPR (Hirata et al., 1994, Zocchi et al., 1993). The deletions were validated by genomic DNA sequencing (Figure S2A). The knockout cells still responded normally to CZ-48 and showed elevated cADPR (Figure 2A, middle bars) and slightly decreased NAD (Figure 2B, middle bars), indicating that neither CD38 nor BST-1 was the target of CZ-48.

We next deleted SARM1 (DNA sequencing see in Figure S2B), which has recently been reported to possess NAD-utilizing activity (Essuman et al., 2017). We obtained a SARM1-knockout HEK-293T cell line (SARM1-KO), which was irresponsive to CZ-48, showing essentially no change in cADPR (Figure 2A, right bars) and NAD (Figure 2B, right bars).

To further prove that SARM1 can actually be activated by CZ-48 and elevate cADPR in cells, we constructed a HEK-293 cell line expressing inducible FLAG-tagged SARM1. As shown in Figure 2C (anti-FLAG, upper panel) the cells expressed progressively more SARM1 when treated with doxycycline (Dox). The induced expression of SARM1 was similar in the presence of CZ-48 (Figure 2C, anti-FLAg, lower panel), indicating that the activation effects of CZ-48 are not on SARM1 expression. Also, controls with CZ-48 alone did not induce SARM1 expression in the absence of Dox (Figure S3A). Figure 2D shows that the intracellular cADPR was increased by CZ-48 (blue line) and was further enhanced by the combination with Dox (purple line). Control cells treated with neither Dox nor CZ-48 showed no cADPR increase (yellow line). Treatment with Dox only also produced no increase (green line, same as the yellow line but obscured by it), indicating that the basal activity of the Dox-induced SARM1 is tightly regulated and requires CZ-48 for activation.

To document the generality of the activation of SARM1 by CZ-48, we treated various cell types and measured cADPR elevation. In addition to human HEK-293T described above, human cervical cancer cell line HeLa, HEK-293 (Figure 2E), rat insulinoma cell line INS-1E, and murine ascites reticulum cell J774A.1 (Figure S3B) were responsive, whereas human promyelocytic leukemia cell line HL-60, human T lymphocytic leukemia cell line Jurkat, human macrophage cell line U937, and human multiple myeloma LP-1 (Figure 2E) were not. The non-responsiveness is related to the low expression of SARM1. As shown in Figure 2F, SARM1 mRNA levels in these cell lines were indeed very low, lower than that in HEK-293, whereas the responsive cells all have higher levels, correlating with the cADPR production in response to CZ-48 stimulation. The mRNA levels did not change after CZ-48 treatment (Figure S3C), consistent with CZ-48 activating the enzymatic activity of SARM1 and not by increasing its expression level.

Not only cell lines but also primary neuronal cells from mouse dorsal root ganglions are responsive to CZ-48. As shown in Figure 2G, cellular cADPR was elevated progressively by CZ-48. After knocking down SARM1 (Figure 2H), the cells became irresponsive to CZ-48 (Figure 2I, right red). These results establish the generality of CZ-48 activating SARM1 to produce cADPR in various cells and that the extents of the cADPR elevation correlate closely with SARM1 expression.

### CZ-48 Mimics Endogenous Metabolite, NMN, in Activating SARM1

**Figure 3:**
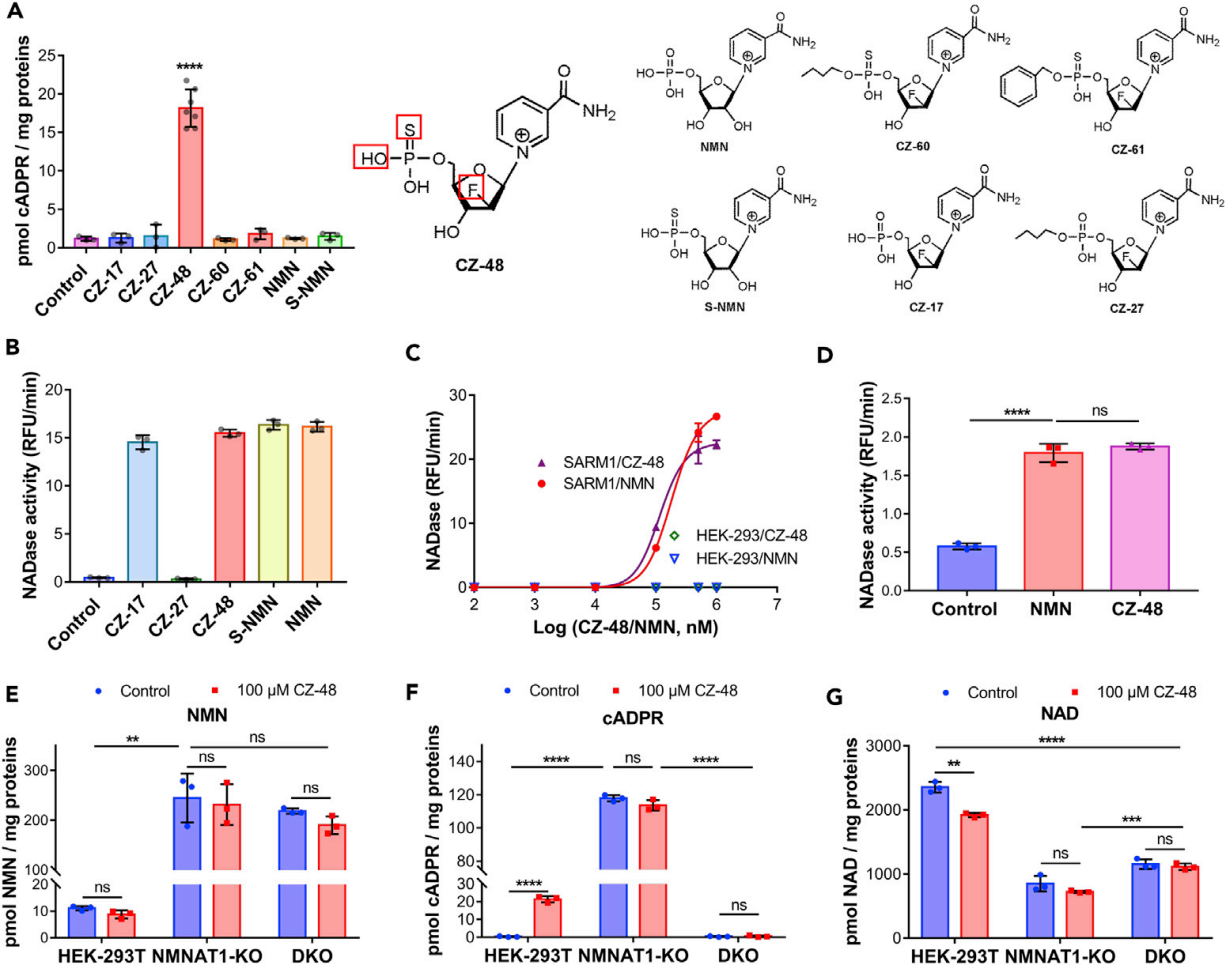
CZ-48 Mimics Endogenous Metabolite NMN in Activating SARM1 (A) HEK-293T cells were treated with 100 μM of different compounds for 24 h, and cADPR contents were measured. The structures of the compounds are shown in the right panel. (B) SARM1-FLAG-overexpressing HEK-293 cells were permeabilized by 100 μM digitonin, and the supernatant was incubated with 100 μM of different compounds, together with 50 μM ɛNAD, and the activities of SARM1 (slopes of the fluorescence production) were analyzed and plotted. (C) The dose-response curves of CZ-48 and NMN on SARM1-FLAG in the cell lysate. Lysate of SARM1-FLAG cells, with HEK-293 as a control, were incubated with different doses of NMN or CZ-48, and NADase activities were measured as (B). (D) The proteins SARM1-FLAg were immunoprecipitated with anti-FLAg beads, eluted with 3× FLAG peptide, and the NADase activities were measured as in (B) in the presence of 100 μM NMN or CZ-48. (E–G) Cellular levels of NMN (E), cADPR (F), and NAD (G) were measured by cycling assays in wild-type, NMNAT1-knockout, and NMNAT1/SARM1 double knockout (DKO) HEK-293T cells. All the above experiments were repeated at least three times (means ± SDs; n ≥ 3; Student's t test, **p < 0.01, ***p < 0.001, ****p < 0.0001).

CZ-48 is a structural mimetic of NMN. In addition to replacing the ribose with 2-deoxy-2-fluoro-D-arabinose, one of the oxygens of the phosphate is substituted with a sulfur (Figure 3A, inset). To identify the structural determinants critical for its activating effect, a series of analogs of CZ-48 were used (Figure 3A). The sulfo-substitution is indispensable, because NMN, CZ-17, and CZ-27 were all inactive. Any additional modification on the phosphate (CZ-60 and CZ-61) also rendered the compound inactive. The compound S-NMN is likewise inactive, indicating that the fluoro-substitution is also critical for the cellular activation. The stringent structural requirement indicates that the binding site for CZ-48 has receptor-like specificity.

Next, we studied the structure-activity relationship *in vitro*. Cell lysates containing SARM1-FLAG were tested for NADase activity using an analog of NAD, etheno-NAD (ɛNAD), which shows increased fluorescence when cyclized to ɛ-cADPR (Graeff et al., 1996) or hydrolyzed to ɛ-ADP-ribose. As shown in Figure 3B, except CZ-27, whose phosphate group was modified by an alkyl chain, all other compounds could activate SARM1 as well as CZ-48. Taking the results shown in Figures 3A and 3B together, it can be concluded that the sulfo-substitution of the hydroxyl of the phosphate group and the fluoro-substitution in the ribose were essential for the cell permeability of CZ-48, enabling it to activate SARM1 in live cells.

As shown in Figure 3C, CZ-48 activated SARM1-FLAG *in vitro* in a concentration-dependent manner (purple triangles) with a half maximal concentration at around 50 μM. NMN activated SARM1 in a very similar manner (Figure 3C, red dots) and with an essentially identical half maximal concentration, which is broadly within its endogenous concentration (Trammell and Brenner, 2013). The lysates of the non-transfected HEK-293 served as a negative control and showed undetectable activity either with NMN (blue triangle) or with CZ-48 (green diamonds, superimposed by blue triangles). The identical activation concentrations of CZ-48 and NMN clearly show that CZ-48 is a true and effective mimetic. Moreover, at the concentrations below saturation, the effects of the two compounds were additive *in vitro*, as shown in Figure S1B, which further substantiated the conclusion.

Although NMN could similarly activate SARM1 *in vitro* as CZ-48, it could not do so in cells, highlighting CZ-48 as a valuable and cell-permeant probe for NMN. We further determined if CZ-48 is innocuous to cell metabolism, by using cells devoid of SARM1 and measuring more than 2,000 metabolites of the cells. All showed high consistency between control and the CZ-48-treated groups (Figure S4A), indicating that CZ-48 (100 μM) has minimal off-target effects, if any, on the cell metabolism.

To determine whether CZ-48 (or NMN) activates SARM1 directly or requiring other factors, the SARM1-FLAG protein was isolated from the cell lysates by immunoprecipitation using anti-FLAG and its activities were tested with ɛNAD. As shown in Figure 3D, SARM1-FLAG after purification could still be activated by NMN and CZ-48. The basal activity was somewhat increased, which may well be caused by the partial release of the auto-inhibitory domain (detailed later in Figure 5) during immunoprecipitation. The data indicate that CZ-48 (or NMN) acts directly on SARM1.

The activation of SARM1 by CZ-48 does not involve covalent modifications but is completely reversible. After removing CZ-48 by centrifugal filtration, the activated SARM1-FLAG was recovered and the activation was tested by adding fresh CZ-48. As shown in Figure S4B, the recovered SARM1-FLAG showed full activation as the untreated protein. The reversibility is notably distinct from its inhibitory action on CD38, which we show by crystallography to involve the formation of covalent linkage of CZ-48 with the catalytic residue (Kwong et al., 2012).

Next, we tested whether endogenous NMN can activate SARM1. Nicotinamide mononucleotide adenylyltransferase 1 (NMNAT1) is the major enzyme catalyzing the formation of NAD from NMN and ATP. By knocking out NMNAT1 in HEK-293T cells (DNA sequencing and western blots see in Figures S2C and S2E, respectively), cellular NMN contents were elevated more than 20-fold (Figure 3E). Concomitantly, cADPR levels increased dramatically (Figure 3F), whereas NAD levels decreased by two-thirds of normal (Figure 3G). Treatment with CZ-48 produced no further increase in cADPR, indicating that SARM1 was fully activated by the endogenous NMN. In NMNAT1/SARM1 double knockout cells (DKO, DNA sequencing in Figure S2D), NMN levels remained at similarly high levels as NMNAT1-KO cells (Figure 3E), whereas the dramatic increase in cADPR levels was eliminated and remained at the basal levels as seen in the SARM1-KO (Figure 3F). The NAD levels in DKO recovered to half of normal (Figure 3G). The above data indicated that high concentration of cellular NMN can fully activate SARM1 to produce cADPR from NAD. The fact that the levels of the three metabolites were not further changed by CZ-48 in both NMNAT1-KO and DKO cells confirms that CZ-48 and the endogenous NMN act on the same target.

Summarizing, the results indicate that CZ-48 mimics cellular NMN in activating SARM1 enzymatically. Its cell permeability makes it remarkably useful. In the following studies, we used CZ-48 to activate SARM1 *in cellulo* and NMN *in vitro*.

### Enzymatic Activities of the Activated SARM1

Hitherto, the only known reaction that can cyclize NAD to cADPR in mammalian cells is that catalyzed by CD38 (Howard et al., 1993), which we have elucidated in detail (Lee, 2006, Liu et al., 2009, Liu et al., 2005, Liu et al., 2008a, Liu et al., 2008b, Zhang et al., 2011). It consists of cleaving the nicotinamide ring and forming an intermediate with Glu226. Subsequent attack by the adenine group results in cyclization and production of cADPR. Attack of the intermediate by water produces ADP-ribose. If nicotinic acid (NA) is present, its attack on the intermediate results in a base-exchange reaction and produces nicotinic acid adenine dinucleotide phosphate (NAADP) (Aarhus et al., 1995, Lee and Aarhus, 1995), which is also a universal Ca^2+^ messenger for mobilizing the endolysosomal stores (Brailoiu et al., 2009, Calcraft et al., 2009). To determine if the same catalytic mechanism is used by SARM1, all these reactions were measured using HPLC.

To prepare large amounts of proteins needed for the assay, we constructed a BC2-tagged (Bruce and McNaughton, 2017) SARM1 with the N-terminal mitochondria localization signal truncated (SARM1-dN) and purified with beads conjugated with BC2 nanobodies (Bruce and McNaughton, 2017). About 700 nM SARM1-dN isolated by the BC2 beads was incubated with 100 μM substrates in the presence or absence of NMN.

**Figure 4:**
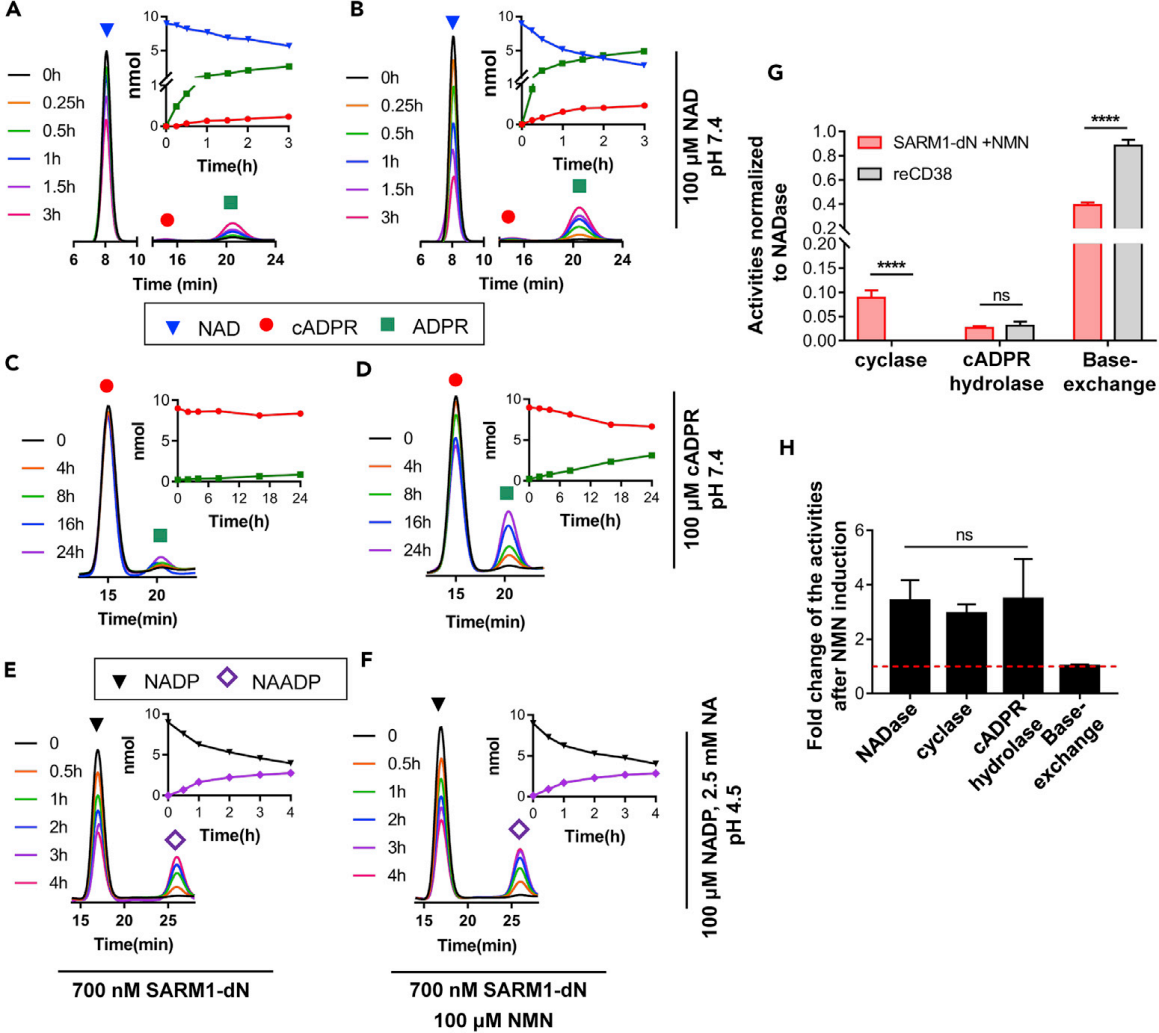
The Enzymatic Activities of SARM1 with or without NMN Activation BC2T-tagged SARM1 with the N-terminal location signal truncated (SARM1-dN, c.f. Figure 5A and Methods) was immunoprecipitated by BC2Nb beads and quantified by western blots. Around 700 nM of SARM1-dN (with or without pre-treatment of 100 μM NMN) was used in three reactions. (A and B) The activities of NAD hydrolase and ADP-ribosyl cyclase. The protein, 700 nM SARM1-dN (A, without NMN; B, with NMN), was incubated with 100 μM NAD in KHM (pH 7.4) for different time periods, and the products were analyzed by HPLC. Insets (above the red dots): enlarged peaks of cADPR in the chromatogram; insets (dot plot): quantification of the products. Blue triangles, NAD; red circles, cADPR; green squares, ADP-ribose. (C and D) cADPR hydrolase activity. Similar reactions were set and analyzed as in (A and B), except the substrate was replaced by the same amount of cADPR. Insets: Red, cADPR; green, ADP-ribose. (E and F) Base-exchange reaction. Similar reactions were set and analyzed as in (A and B), except the substrate was replaced by same amount of NADP and 2.5 mM NA in 15 mM acetate buffer (pH 4.5). Insets: black, NADP; purple, NAADP. (G) The activities of NMN-activated SARM1-dN (in B, D, F) and reCD38 (Graeff et al., 2001) were normalized with their NAD hydrolase activities. (H) The activities including NADase (ADPR production rate in A and B), ADP-ribosyl cyclase (cADPR production rate in A and B), cADPR hydrolase (ADPR production rate in C and D), and base-exchange activities (NAADP production rate in E and F) were calculated and presented as the fold change after NMN induction. The red dashed line is the activity level of SARM1-dN without NMN. The experiments were repeated at least three times. All the above experiments were repeated at least three times (means ± SDs; n = 3; Student's t test, ****p < 0.0001).

As shown in Figure 4A, SARM1 possessed NADase activity, hydrolyzing NAD (blue triangles) to ADP-ribose (green squares). Activation by NMN stimulated the NADase activity and accelerated the decrease of NAD (Figure 4B, blue triangles), which also stimulated the cyclization of NAD to produce cADPR (cyclase activity) by 2- to 3-folds (Figures 4A and 4B, red circles, and Figure 4H). Likewise, NMN also stimulated the cADPR-hydrolyzing activity of SARM1 (Figures 4C, 4D, and 4H). SARM1 also possessed a base-exchange activity similar to reCD38, exchanging the nicotinamide group of NADP with NA to produce NAADP (Figures 4E and 4F). The fact that SARM1, like reCD38, can use three different substrates, NAD, cADPR, and NADP, and produce three different products, cADPR, ADPR, and NAADP, strongly suggests that its catalytic mechanism is the same as that of reCD38.

SARM1 is, however, a much slower enzyme than reCD38. The specific NADase activity of SARM1 was measured to be 1.16 ± 0.26 mol/mol enzyme/min, which was, however, much slower than 2.59 ± 0.17 kmol/mol enzyme/min measured for reCD38 (Graeff et al., 2001). To be able to quantitatively compare the three activities of SARM1 and reCD38, we normalized each activity to the NADase activity of each enzyme. As shown in Figure 4G, SARM1 showed much greater relative cyclase activity than reCD38, which mainly catalyzed NAD hydrolysis and cADPR production was almost undetectable (Figure S5A). The relative cADPR hydrolase and the base-exchange activities of the two enzymes were similar. An enzymatically inactive mutant of SARM1 (E642A) (Essuman et al., 2017) was used as a control, which did not show any activity for all the reactions (Figure S5D–S5G). The greatly enhanced cyclase activity of SARM1 relative to its other activities, especially after NMN activation, makes it much more effective in elevating cellular cADPR than CD38. In contrast to SARM1, recombinant CD38 (reCD38) was inhibited by both NMN (Figure S5H) and CZ-48 (IC_50_ around 10 μM, Figure S5H, green triangles).

Notably, NMN stimulated the NADase, cyclase, and cADPR hydrolase activities all by a similar 3- to 4-folds (Figure 4H). That all three activities are stimulated to a similar extent is consistent with the stimulation being affected by relieving an auto-inhibition, unmasking all three activities possessed intrinsically by SARM1. This will be described in more details below. The base-exchange activity was detected without activation by NMN and was not further stimulated by it. This is likely because the acidic assay condition already caused the release of the auto-inhibition.

### CZ-48 and NMN Induce Allosteric Conformational Changes to Activate SARM1

**Figure 5:**
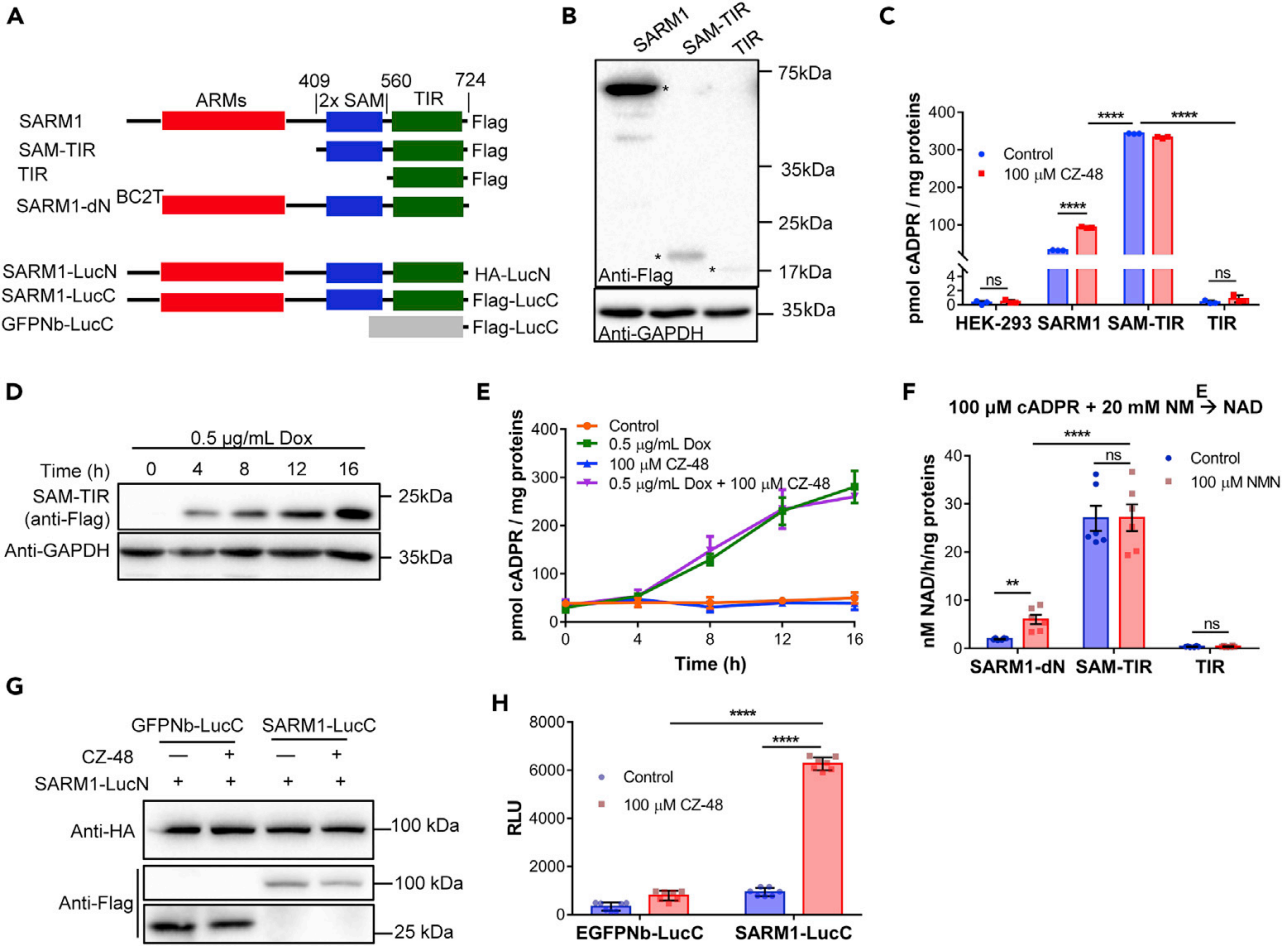
CZ-48 and NMN Induce Allosteric Conformational Changes of SARM1 Leading to its Activation (A–H) (A) Diagram of tagged full-length SARM1 and various truncates (for B–F) and the fusion proteins with the luciferase fragments (last three constructs, for G and H). For the sake of brevity the tags FLAG or HA are omitted in this figure unless otherwise specified. (B) HEK-293 cells stably expressing SARM1 and truncates were constructed. SARM1 and TIR were in constitutive expression cassettes, whereas SAM-TIR was in an inducible expression cassette to prevent cell death caused by overexpression of SAM-TIR. The expression levels of the constitutive SARM1 and TIR, and SAM-TIR induced by 0.5 μg/mL Dox for 20 h, were tested by western blots. Asterisks point to the specific bands. (C) Cells from (B) were treated with 100 μM CZ-48 for 8 h, and the cADPR contents were measured. (D and E) HEK-293 cells carrying an inducible expression cassette of SAM-TIR were treated with 100 μM CZ-48 or 0.5 μg/mL Dox, and protein levels were measured by western blots (D) or cADPR levels (E). (F) Proteins were immunoprecipitated and the cyclase activity *in vitro* was tested by reverse cycling assay with or without the presence of 100 μM NMN. (G and H) HEK-293 cells, co-transfected with the vectors encoding SARM1-LucN/SARM1-LucC or SARM1-LucN/GFPNb-LucC, as a negative control, were treated with 100 μM CZ-48 for 12 h. The expression of fusion proteins (G) and reconstituted luciferase activities (H) were measured by western blots or luciferin incubation reaction, respectively. All the above experiments were repeated at least three times (means ± SDs; n = 3; Student's t test, **p < 0.01, ****p < 0.0001).

SARM1 possesses multiple domains, including an N-terminal domain with multiple armadillo repeat motifs (ARMs), two tandem sterile alpha motif (SAM) domains, and a C-terminal Toll-interleukin-1 receptor (TIR) domain (Figure 5A). It has been proposed that the ARM domain is auto-inhibitory in promoting axonal degeneration, whereas the SAM domain is responsible for mediating the dimerization of the TIR domains that is necessary for the biological activity (Gerdts et al., 2013, Gerdts et al., 2015, Summers et al., 2016).

To determine if the activation of SARM1 by CZ-48 correlates with the biological effects, we first measured the cADPR-producing activities of various domains of SARM1. We constructed and expressed the full-length SARM1 and its two truncates, SAM-TIR and TIR (Figure 5B), in HEK-293 cell lines. The full-length SARM1 and the TIR were constitutively expressed, whereas the SAM-TIR construct was under Dox control.

**Figure 6:**
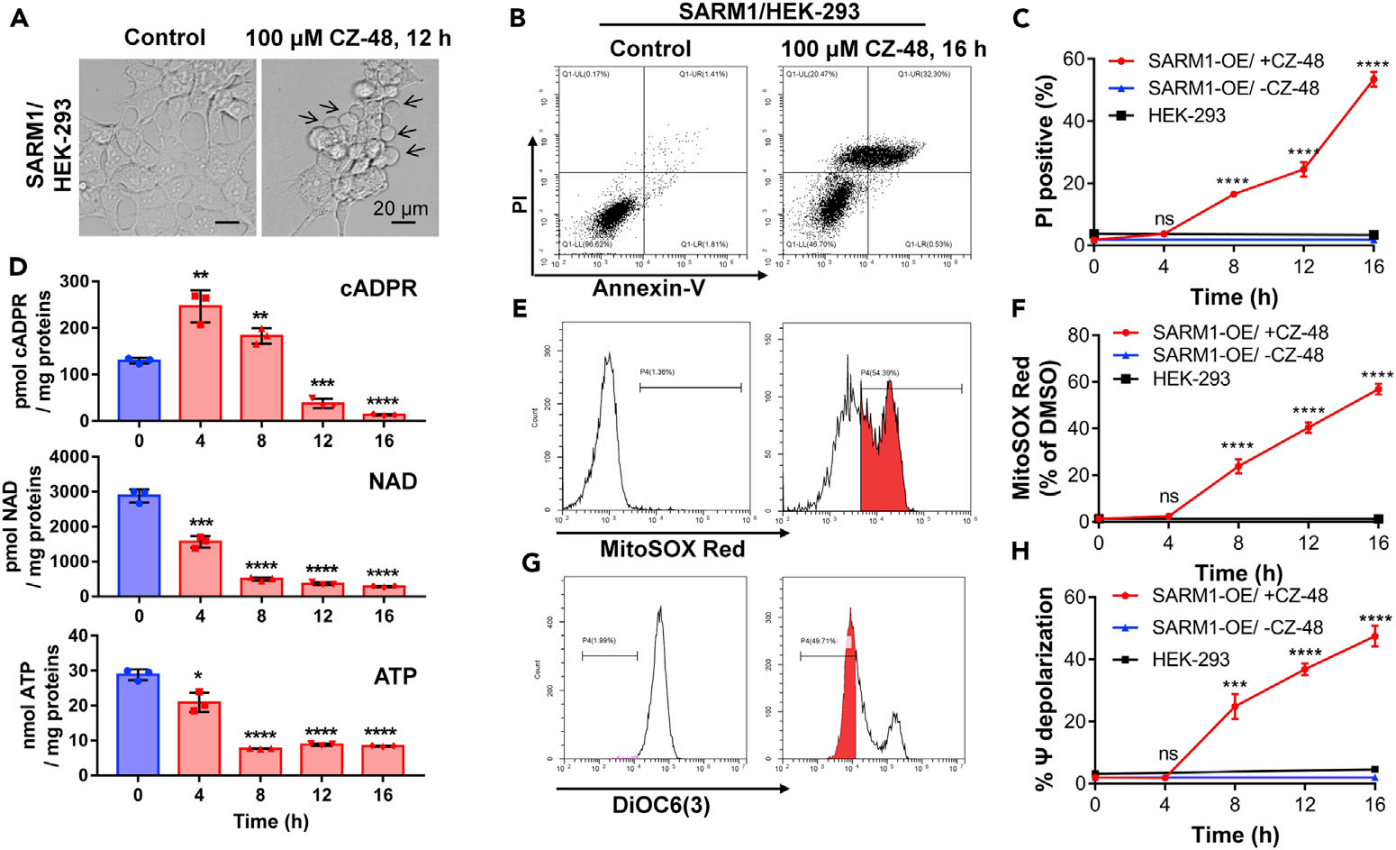
Activation of SARM1 by CZ-48 Induced Cell Death (A–H) Wild-type and SARM1-overexpressing HEK-293 cells were treated by 100 μM CZ-48 for labeled time periods. (A) CZ-48 treatment induces cell blisters (black arrows) and shrinkage in cells overexpressing SARM1. (B and C) Cell viabilities were analyzed by annexin-V/PI staining combining flow cytometry (B), and the PI positive rates of all samples were plotted (C). (D) The cellular contents of cADPR (upper chart), NAD (middle chart), and ATP (lower chart) were measured by cycling assay or luminescent ATP detection assay, as described in Methods. (E and F) Mitochondrial reactive oxygen species contents were measured by MitoSOX red staining and analyzed by flow cytometry (E). The positive rates of all samples were plotted (F). (G and H) Mitochondrial membrane potential was analyzed by DIOC6(3) staining and analyzed by flow cytometry (G). The positive rates of all samples were plotted (H). All the above experiments were repeated at least three times (means ± SDs; n = 3; Student's t test, **p < 0.01, ***p < 0.001, ****p < 0.0001).

As shown in Figure 5C, expression of SAM-TIR after 20 h of Dox treatment resulted in dramatic increases in cellular cADPR, three to four times higher than that measured in cells expressing full-length SARM1. The increase was even more dramatic considering that the amount of SAM-TIR expressed was much less than that of SARM1 (Figures 5B and 5C). The high cADPR levels in SAM-TIR-expressing cells were not sensitive to further activation by CZ-48 (Figure 5C). In contrast, CZ-48 induced further increase in the cADPR levels in SARM1-expressing cells (Figure 5C). The extent of this stimulation was time dependent, and the time course is shown in Figure 6D (upper graph).

The full time course of the SAM-TIR induction is shown in Figure 5D. Concomitant with the increase in SAM-TIR expression, the levels of cADPR also progressively increased (Figure 5E, green squares). Treatment with CZ-48 together with Dox produced no further increase in cADPR (Figure 5E, purple triangles). This is in direct contrast to that observed during Dox-induced expression of full-length SARM1 shown earlier in Figure 2D, where CZ-48 produced substantial enhancement of the cADPR accumulation. Control cells without Dox induction showed no elevation of cADPR levels, with (Figure 5E, blue triangles) or without treatment with CZ-48 (Figure 5E, orange dots). These results indicate that SAM-TIR is constitutively active in producing cADPR even without activation by CZ-48.

The constitutive activity of SAM-TIR was further substantiated by *in vitro* measurements. SAM-TIR was immune purified from the cell lysates (Figure S6), and its reverse cyclase activity was measured. Figure 5F shows that SAM-TIR was fully active and its activity was not further stimulated by NMN. In contrast, immunopurified SARM1 was sensitive to activation by NMN (Figure 5F, SARM1-dN). The results are consistent with the ARM domain in SARM1 being self-inhibitory, and its deletion rendered SAM-TIR constitutively active in producing cADPR. The SAM domain is necessary for the full activity because expression of just the TIR domain produced very little cADPR (Figure 5F, TIR).

Previous studies suggest that dimerization of the catalytic domain of SARM1 is required for the damage-induced axonal degeneration (Gerdts et al., 2015). We employed the protein-fragment complementation assay (PCA) to determine if CZ-48 can induce similar conformational changes in SARM1. Fragments of luciferase (Liu et al., 2017) (hemagglutinin [HA]-LucN and FLAG-LucC) were each fused to the C terminus of SARM1 (Figure 5A). Dimerization of SARM1 at its C-terminal domain would reconstitute the luciferase and produce luminescence. As a negative control, one of the fragment, FLAG-LucC, was fused to an irrelevant protein, a nanobody of GFP (GFPNb-LucC) (Liu et al., 2017). The PCA probes, either SARM1-LucN and SARM1-LucC or SARM1-LucN and GFPNb-LucC, were transiently transfected in HEK-293 cells. The expression of the PCA proteins was confirmed by western blots (Figure 5G) using anti-HA to detect SARM1-LucN, and anti-FLAG for SARM1-LucC and GFPNb-LucC. After treating with 100 μM CZ-48, the cell line expressing the two SARM1 PCA probes produced much higher luminescence than the control cell line expressing SARM1-LucN and GFPNb-LucC (Figure 5H), indicating that CZ-48 treatment did induce dimerization of the C-terminal TIR.

### Activation of SARM1 by CZ-48 Induced Non-apoptotic Cell Death

SARM1 has been reported to be an executioner of axon degeneration (Gerdts et al., 2015) and cell apoptosis (Panneerselvam et al., 2013). It has also been shown to induce a particular type of non-apoptotic cell death termed SARMoptosis (Summers et al., 2014). To demonstrate the functional utility of the cell-permeant CZ-48, we measure its effectiveness in inducing non-apoptotic cell death. We constructed a HEK-293 cell line stably expressing full-length SARM1 without any tag, which showed expected mitochondrial localization (Figure S7A–S7C), with the correct molecular weight, and in mitochondrial fractions (Figure S7D). The morphology of the cells was normal (Figure 6A, left picture), and they proliferated similar to wild-type HEK-293 cells (Figure S7E), indicating that the unactivated SARM1 itself is innocuous. However, when treated with CZ-48, the cells progressively shrank and blistered (Figure 6A, right picture, arrows). Cell death was measured by the double staining with annexin-V and propidium iodide (PI). As shown in Figures 6B and S7F, the typical apoptosis feature, annexin V-single-positive population, was not observed in the flow cytometry diagram, which was further supported by the negative results of caspase-3 activity tests (Figures S7G and S7H). However, quantification of PI-positive rate showed that non-apoptotic cell death significantly increased at 8 h post-treatment and reached 60% after 16 h (Figure 6C).

Figure 6D (upper chart) shows that the expression of SARM1 led to cADPR accumulation (compare with those of HEK-293 cells, c.f. Figure 5C), whose levels were further increased by CZ-48 (4 h). At 8 h after CZ-48 treatment, non-apoptotic cell death commenced as indicated by PI staining (Figure 6C) and the cADPR levels fell, probably due to cell leakage. Concomitant with cADPR elevation, NAD and ATP levels gradually decreased (middle and lower charts). CZ-48 also induced mitochondrial dysfunctions as reflected in increased superoxide production (MitoSOX red staining, Figures 6E and 6F) and mitochondrial depolarization (Figures 6G and 6H), with time courses similar to those seen in PI staining. These results clearly establish the effectiveness and utility of CZ-48 as a modulator of SARM1 and its biological functions in live cells.

It should be noted that CZ-48 is totally innocuous in cells expressing only minimal SARM1 (HEK-293, c.f. Figure 2F). Cell death, superoxide release, or mitochondrial depolarization was not observed even after 16 h of treatment with CZ-48 (black squares, Figures 6C, 6F, and 6H). An additional control shows that without activation by CZ-48, even cells overexpressing SARM1 were normal with no signs of cell deterioration (blue triangles, Figures 6C, 6F, and 6H). The results indicate that the CZ-48-induced non-apoptotic cell death requires sufficiently high amounts of the activated SARM1 in the cells and is likely caused by the levels of metabolic activities produced by it.

## Discussion

**Figure 7:**
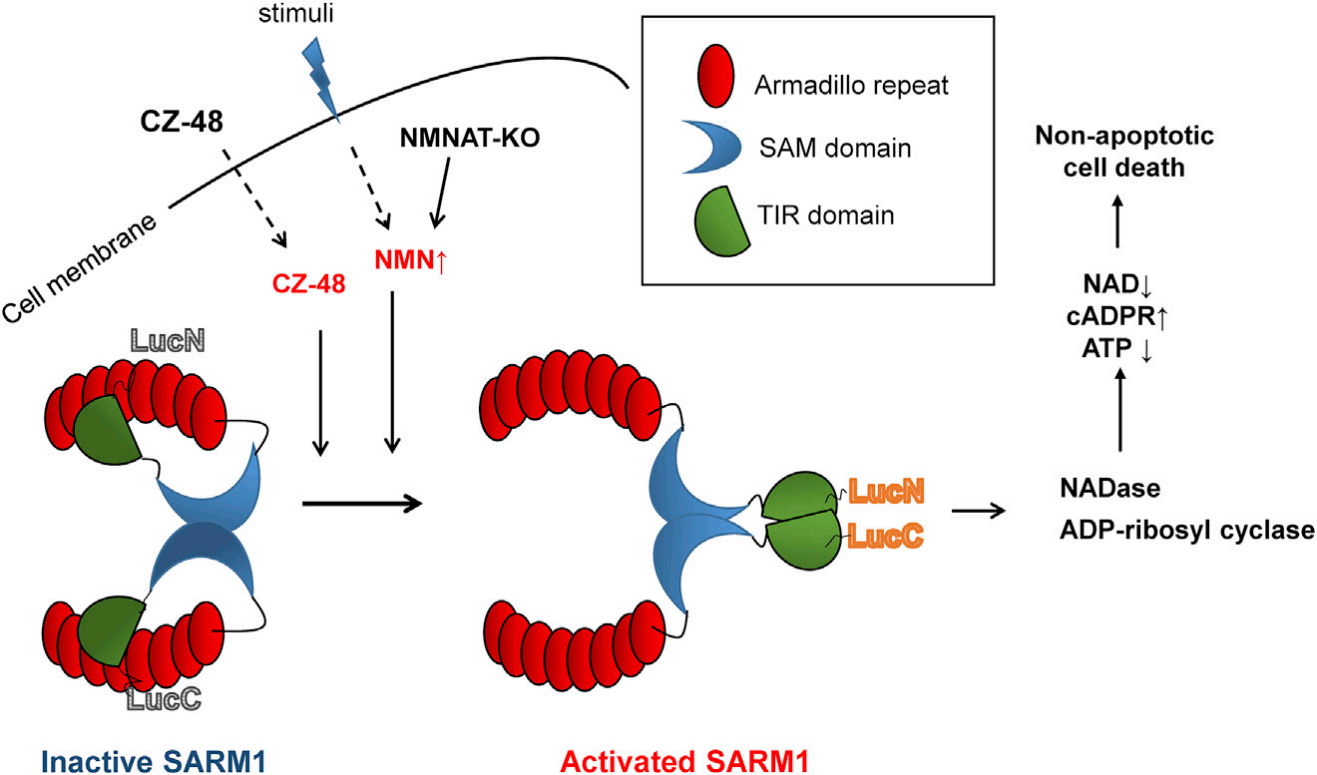
Summary of the Activation of SARM1 by CZ-48 and NMN

The scheme depicted in Figure 7 summarizes the results described in this study on the mechanism of SARM1 activation triggered by CZ-48. SARM1 is a large molecule.

The repeating motifs in the SAM and ARM domains have the propensity to interact and could result in forming constitutive SARM1 dimers. The PCA results (Figure 5H) showing minimal luminescence indicate that the TIR domains are far apart. In this resting state, the enzymatic activities are self-inhibited by its N-terminal ARM domain, suggesting that the C-terminal SAM-TIR domain may fold back and be secluded by the ARM domain. This folded conformation prohibits the TIR domains from dimerization. Extracellular addition of CZ-48, or elevated cellular NMN, induces an allosteric conformational change in SARM1. The unfolding of the ARM domain releases the catalytic TIR domains, allowing them to dimerize and activate the enzymatic activities. We have used PCA (LucC, LucN) in this study to directly monitor this conformation change. The activation by CZ-48 also leads to progressive accumulation of cellular cADPR in a wide range of human and mouse cell lines as well as primary cells. The extents of cADPR accumulation vary widely among the cells because the accumulation not only is time dependent but also depends on the endogenous levels of SARM1.

The results presented in this study establish SARM1 as a cADPR-producing enzyme regulatable by an endogenous metabolite, NMN, and its mimetic, CZ-48. A functional consequence of the activation of SARM1 is the induction of non-apoptotic cell death. SARM1 thus possesses all the characteristics of a signaling enzyme and is a potentially important effector of intracellular calcium changes.

It is remarkable that SARM1 and CD38, two entirely different proteins with no sequence similarity, can use the same substrates, NAD and NADP, and catalyze the same multiple activities, cyclization, hydrolysis, and base exchange. These were documented in detail in this study using immunopurified SARM1. The catalytic mechanism of SARM1 is thus likely to be the same as that of CD38, which we have fully elucidated by crystallography and mutagenesis (Liu et al., 2005, Liu et al., 2008b). Compared with CD38, SARM1 is much more efficient in cyclizing NAD to cADPR, making it even more proficient in elevating cADPR in cells. Indeed, in HEK-293 cells, whose endogenous SARM1 is so low that it is detectable only by PCR but not by western blots, CZ-48 can still readily elevate cADPR, albeit requiring longer accumulation time (Figure 2E). The effectiveness of SARM1 in producing cADPR in cells thus depends on its expression levels and on whether it is fully activated.

Our results show that both CZ-48 and NMN can induce the same conformational changes in SARM1 and activate its enzymatic activities. However, NMN can do so only in cell lysates but not in intact cells (Figures 3A and 3C), indicating it is not permeable to the cells we tested. The cell-permeant characteristic of CZ-48 thus offers an advantage as a tool for manipulating SARM1's activity *in vitro* and *in vivo*. The fact that CZ-48 can activate SARM1 at the same concentrations as NMN and that it is innocuous in cells not expressing sufficient SARM1 (Figures 6C, 6F, 6H, S7E, and S7F) indicates that it is a true mimetic with essentially no detectable off-target effect.

The cell impermeability of NMN in various cells has greatly hampered the investigation on its biological functions. Although NMN is a principal intermediate of NAD synthesis, how it is taken up by cells has not been fully elucidated. It was reported that the extracellular NMN could accelerate the axonal degeneration induced by axotomy, indicating that NMN might be permeable to the neurites. Furthermore, long-term administration of NMN has been shown to mitigate the age-associated physiological decline in mice (Mills et al., 2016) and the group recently has documented that Slc12a8, highly expressed in the mouse small intestine, specifically transports NMN (Grozio et al., 2019). In other cells, it has also been proposed that NMN is first converted to nicotinamide riboside by ectonucleotidases, such as CD73 (Garavaglia et al., 2012), which can then be taken up by nucleoside transporters (Nikiforov et al., 2011, Ratajczak et al., 2016). These uncertainties in uptake of NMN can be bypassed by CZ-48, which can readily permeate the cell membrane to directly activate the endogenous SARM1 and affect its biological functions.

Most studies on SARM1 focus on its role in axonal degeneration. This is mainly because SARM1 is highly expressed in the nervous system (Chen et al., 2011) and its activation leads to depletion of NAD and neuronal death. Here we show that SARM1 is endogenously present in diverse human and murine cells, in some cases, at very low levels. Its activation in these cells would make minimal changes in the tightly regulated NAD contents (c.f. Figure 2B) but can produce large increase in cADPR level. This suggests that the function of SARM1 in non-neuronal cells may not be for inducing cell death, but instead, may serve as a Ca^2+^-signaling enzyme. In fact, Ca^2+^ changes were observed in the SARM1-mediated axonal degeneration (Loreto et al., 2015). Cell death is likely the cumulative result of many factors resulting from SARM1 activation, and cADPR may be one of them.

SARM1 indeed possesses several characteristics fitting for functioning as a Ca^2+^-signaling enzyme like CD38. First, it is auto-inhibited in resting but can be readily activated by signaling molecules, such as NMN and also kinases (Murata et al., 2018). Second, its catalytic domain faces the cytoplasm (Gerdts et al., 2013) with full access to the substrate, NAD^+^. Last but not least, compared with CD38, the activated SARM1 has higher cyclase activity, allowing it to elevate cADPR more efficiently than CD38. In many circumstances, the cellular cADPR levels could serve as a better indicator for SARM1 activation than the cellular NAD contents and cell death.

Ever since the discovery of cADPR, investigation on its biosynthesis has been focused on CD38. Its catalytic mechanism is now well understood at the molecular level. Recent results have established the existence of the type III CD38 (Liu et al., 2017, Zhao et al., 2012) inside cells, in addition to the type II CD38 on cell surface (Liu et al., 2017, Zhao et al., 2012, Zhao et al., 2014). Modulation of cellular cADPR levels is affected by interactions of the type III CD38 with the cytosolic regulator, CIB1. In this study, we establish another totally different mechanism for producing cADPR in cells by SARM1, which is directly regulated by NMN. This points to a hitherto unexplored relationship between Ca^2+^ signaling and NAD metabolism. The cell-permeant activator of SARM1, CZ-48, described here should provide a valuable and convenient tool to address this issue. Last but not least, the differential and selective effects of CZ-48 on SARM1 and CD38, activating the former and inhibiting the latter (Kwong et al., 2012), make it a perfect probe to investigate the biological functions of SARM1 without the interference from the CD38 pathway.

### Limitations of the Study

In this study, we synthesize and characterize CZ48, a cell-permeant mimetic of NMN, and show that it can activate SARM1 to produce cADPR from NAD. Although we have tested its effectiveness in a number of different cell types, its generality needs to be further demonstrated, especially in neurons, where SARM1 is known to be a key regulator in axonal degeneration. It is also important to further increase its efficacy and permeability, so as to enhance its therapeutic potential as a modulator of NAD metabolism and an activator of cADPR synthesis. The detailed mechanisms of SARM1 activation and regulation also need further investigation.

## Methods

All methods can be found in the accompanying Transparent Methods supplemental file.

## Supplemental Information

Document S1 . Transparent Methods and Figures S1–S7
