# Supplementary material for: A Cell-Permeant Mimetic of NMN Activates SARM1 to Produce Cyclic ADP-Ribose and Induce Non-apoptotic Cell Death
Source: iScience. 2019 May 4;15:452–66. doi: 10.1016/j.isci.2019.05.001 (PMC6531917; doi:10.1016/j.isci.2019.05.001)
Supplement: Document S1 — . Transparent Methods and Figures S1–S7 [file mmc1.pdf]

ISCI, Volume *15*

Supplemental Information

A Cell-Permeant Mimetic of NMN Activates SARM1 to Produce Cyclic ADP-Ribose and Induce Non-apoptotic Cell Death

Zhi Ying Zhao, Xu Jie Xie, Wan Hua Li, Jun Liu, Zhe Chen, Ben Zhang, Ting Li, Song Lu Li, Jun Gang Lu, Liangren Zhang, Li-he Zhang, Zhengshuang Xu, Hon Cheung Lee, and Yong Juan Zhao

Supplementary Information

Transparent Methods
Figure S1
Figure S2
Figure S3
Figure S4
Figure S5
Figure S6
Figure S7
Reference

Transparent Methods

Reagents and antibodies

Lipofectamine^{TM} 2000, Dulbecco's Modified Eagle Medium (DMEM), Iscove's modified Dulbecco's medium (IMDM), RPMI 1640 Medium (1640), trypsin, fetal bovine serum (FBS), penicillin/streptomycin solution, and Alexa Fluor-conjugated donkey anti-mouse or anti-rabbit IgG were purchased from Life Technology. Polyclonal anti-SARM1 were prepared and purified against the recombinant SARM1 by Absea Biotechnology Ltd; Mouse anti-Flag (F1804-1MG) was obtained from Sigma–Aldrich; Rabbit anti-flag (20543-1-AP) and anti-HA (51064-2-AP) were obtained from Proteintech; Anti-Tom20 (sc-17764) was obtained from Santa Cruz Biotechnology; Anti-GAPDH (D110016-0200) was obtained from Sangon Biotech. NADase was prepared from *Neurospora crassa*. NAD, nicotinamide, digitonin, poly-L-lysine, propidium iodide, 4′,6-diamidino-2-phenylindole dihydrochloride (DAPI), Anti-FLAG M2 Magnetic Beads, 3× FLAG peptide, compounds used in the cycling assay(Graeff & Lee, 2002) and sea urchin egg homogenate assay(Clapper, Walseth et al., 1987) were obtained from Sigma-Aldrich. Anti-HA Magnetic Beads (88837) was obtained from Thermo Scientific. Protein G Sepharose 4 Fast Flow (17-0618-01) and NHS-activated Sepharose 4 Fast Flow (17-0906-01) were obtained from GE Healthcare. 3,3'-Dihexyloxacarbocyanine iodide (318426) was purchased from MedChemExpress. MitoSOX Red (40778ES50) was obtained from YESEN.

Constructs

The plasmids pCDH-EF1-T2A-Puro and pCDH-EF1-IRES-Neo were from System Biosciences. The plasmids pLenti-puro (#39481), pENTR1A-GFP-N2 (#19364), pInducer20 (#44012), pSpCas9(BB)-2A-Puro (PX459, #48139), psPAX2 (#12260), pMD2.G (#12259) were from Addgene. The CDS (Coding DNA Sequence) of SARM1 gene was synthesized according to the reference sequence in GeneBank (NM_015077.3) by Vigene Bioscience (Shangdong, China). pCDH-NbGFP-LucC was constructed by Dr. J. Liu(Liu, Zhao et al., 2017). The fragments of BC2T-TEV were synthesized by Sangon Biotech and inserted into PENTR1A by the restriction enzymes.

To construct the pLenti-SARM1-Flag, -SAM-TIR-Flag, -TIR-Flag, -BC2T-TEV-SARM1-dN, or pInducer20-SARM1, -SAM-TIR-flag, the inserts were amplified by PCR from the synthesized SARM1 DNA, inserted into pENTR1A by the restriction enzymes designed in the primer sequences and transferred to pLenti-puro or pInducer20 by LR reaction.

A small hairpin RNA (shRNA) interference vector targeting murine SARM1 was produced by annealing oligonucleotides containing the targeting sequence (5'-CTGGTTTCTTACTCTACGAAT -3') and a loop sequence into a lentiviral vector, pLKO.1 (no. 10878; Addgene), and pLKO.1-scramble (no.1864; Addgene) as a control.

To construct the pCDH-SARM1, SARM1 was amplified by PCR and subcloned to pCDH-EF1-IRES-Neo. To construct pCDH-SARM1-LucN and -SARM1-LucC, the fragments of SARM1 and LucN or LucC(Lee & Aarhus, 1991) were amplified by PCR and subcloned into pCDHEF1-LucN-T2A-Puro or pCDH-EF1-LucC-IRES-Neo, respectively.

All primers used in the above cloning were listed in the following table.

Proteins to express	Destination vectors	Primers
SARM1-Flag	pENTR1A, pLenti-puro, pInducer20	5'-**AAGCTT**ATGGTCCTGACGCTGC-3'<br>5'-**GGTACC**TTATTTGTCATCGTCATCCTTG-3'
SAM-TIR-Flag	pENTR1A, pLenti-puro, pInducer20	5'-**AAGCTT**ATGATCCTGCCCTCCGTG-3'<br>5'-**GGTACC**AAGGTTGGACCCATGGGT -3'
TIR-Flag	pENTR1A, pLenti-puro	5'-CCG**AAGCTT**ATGACTCCAGATGTCTTC -3'<br>5'-**GGTACC**AAGGTTGGACCCATGGGT-3'
BC2T-TEV-SARM1-dN	pENTR1A, pLenti-puro	5'-**GGTACC**CTGGCGGTGCCTGGGCCAG-3'<br>5'-**GCGGCCGC**CTAGGTTGGACCCATGG-3'<br>5'-**AAGCTT**ATGCCAGACAGAAAAGCGG-3'<br>5'-**GGTACC**ACCCTGAAAATACAAATTC-3'
SARM1	pCDH-EF1-MCS-IRES-neo	5'-**TCTAGA**GCCACCATGGTCCTGACGCTGCTTC-3'<br>5'-**GAATTC**TTAGGTTGGACCCATGGGTG-3'
SARM1-LucN	pCDH-EF1-MCS-T2A-puro	5'-**TCTAGA**GCCACCATGGTCCTGACGCTGCTTC-3'<br>5'-**GCTAGC**GGTTGGACCCATGGGTGCAG-3'<br>5'-**GAATTC**TACCCATACGATGTTCCAGATTAC-3'<br>5'-TAT**GGATCC**GCCTATGCCGCCCTGTGC-3'
SARM1-LucC	pCDH-EF1-MCS-IRES-neo	5'-**TCTAGA**GCCACCATGGTCCTGACGCTGCTTC-3'<br>5'-**GCTAGC**GGTTGGACCCATGGGTGCAG-3'<br>5'-**GAATTC**GACTACAAAGACGATGACGACA-3'<br>5'-**GGATCC**TTAGTCACCACCGGCCCCCT-3'

Cell culture

All the cell lines, except LP-1 were from ATCC. HEK-293, HEK-293T, HeLa were cultured in DMEM. U937, HL-60, Jurkat, J447A.1, and INS-1E cells were maintained in RPMI 1640. LP-1 cells, gifted by Annie An (School of Pharmaceutical Sciences, Peking University) and verified by STR profiling test before experiments, were cultured in IMDM. All media were supplemented with 10% FBS and 1 % penicillin/streptomycin solution. All the cells were

maintained in a standard humidified tissue culture incubator at 37 °C with 5% CO_2.

DRG neurons were isolated from newborn mouse and cultured as previously described(Chen, Stevens et al., 2008, Sleigh, Weir et al., 2016). The neuronal cells were seeded on plates pre-coated with poly-D-Lysine (Sigma-Aldrich) and laminin (Life Technologies) and were maintained in Neurobasal media (Invitrogen). Neurobasal medium supplemented with 2% B27 (Invitrogen) and 50 ng/mL NGF (2.5S; Harlan Bioproducts), 1 μM 5-fluoro-2'-deoxyuridine (Sigma), and 1 μM uridine (Sigma). Every two days, 50% of the culture medium was replaced. Media containing 100 μM CZ-48 was substituted after the neuronal cells were attached and the cell morphology was monitored daily under microscope. Chinese Kun Ming mouse were obtained from Guangdong Medical Laboratory Animal Center and used under the direction of the Institutional Animal Care and Use Committee (IACUC) at Peking University Shenzhen Graduate School.

Transient transfection and construction of stable cell lines

Transient transfection of HEK-293 or 293T cells was done by Lipofectamine 2000™ according to the manufacturer's instructions. To construct CD38-KO HEK-293T cell line, TALEN pairs targeting exon 3 of CD38 gene were designed (CCAGCGGGACATGTTCaccctggaggacacgctGCTAGGCTACCTTGCTG), in which the spacer region (in lowercase, the predicted DSBs) spans the active site of CD38, Glu226, was cloned into pCS2-eTALEN-T (Viewsolid Biotech, China) and transfected HEK-293T cells. Single-cell colonies were generated by serial dilution and the gene knockout was validated by genomic DNA sequencing following PCR amplification by the primers, 5'-CACACAGAAATCATTGATGCTTAC-3' and 5'-GCTGGTACCCTACTTCTTGTACAG-3'. To construct CD38/BST-1 double knockout cell line, sgRNA (5' TAGTATTCCAAGGATAGTTC-3'), targeting exon 5 of BST-1 gene, was clone into pCAG-T7-Cas9-pgk-Puro-T2A-GFP (Viewsolid Biotech, China) and transfect CD38-KO HEK-293T cells. Single-cell colonies were obtained and validated with the same strategy as above (primer for DNA amplification: 5'- CCACTGCAGTTTCGAGGTTC-3' and 5'-CGCATGAGATGGGGACTAGG-3').

To construct SARM1 knockout HEK-293T cell line, sgRNA targeting exon 8 of SARM1 (5′- ATTGTGACTGCTTTAAGCTG -3′, close to the putative enzymatic site: E642) was subcloned to pSpCas9(BB)-2A-Puro (PX459, #48139, Addgene). HEK-293T cells were transfected with the resulted plasmid and single-cell colonies were obtained and validated with the same strategy as above (primer for DNA amplification: 5′-TATTACACTACAAGGGTTAAGGT-3′ and 5′-TTCAGAAAGGACGATGGAAATG-3′).

To construct the NMNAT1-KO cell line, the sgRNA for CRISPR interference (5' ATGATGACCCGGTGATAGGC-3'), targeting on exon 2 of NMNAT1 gene, was clone into an all-in-one CRISPR plasmid, pSpCas9(BB)-2A-Puro and transfect into HEK293T cell line. After transfection, single-cell colonies were prepared by serial dilution. The validation of KO candidates was carried out by western blot with anti-NMNAT1 antibody (sc-271557, Santa cruz) and genomic DNA sequencing by PCR amplification using the primer 5'-ATCTAGGAAGGTACACAGTTGTCAAAGG-3' and 5'-CATAATGAAAGACACAGAGGGGCTAAGG-3'.

To construct NMNAT1/SARM1 double knockout HEK-293T cell line, vectors

expressing the sgRNA targeting exon 8 of SARM1 were transfected to NMNAT1-knockout HEK-293T cells. The single-cell colonies were obtained and validated with the same strategy as above.

To construct cell lines stably expressing protein-of-interest, the lentiviral particles were prepared by transfecting HEK-293T cells with the corresponding lentivectors, pMD2.G and psPAX2, followed by cell infection and selection of HEK-293 or HEK-293T cells by the corresponding antibiotics as described previously(Liu et al., 2017).

Chemical synthesis

The synthesis route of the compounds CZ-17, CZ-27, CZ-48, CZ-60 and CZ-61 has been described in the previous report(Kwong, Chen et al., 2012).

To synthesize S-NMN, nicotinamide riboside (NR, 200 mg, 60 mmol) in $PO(OMe)_3$ (2.0 mL) at 0 °C was added to $PSCl_3$ (0.45 mL, 300 mmol). The mixture was stirred at the same temperature and the reaction was monitored by LC-MS until all NR was consumed. The reactant was neutralized with 1 N NaOH at 0 °C to pH 7.0, filtered and concentrated in *vacuo*. The product was purified by HPLC using YMC-Pack ODS AQ column (250 mm × 10 mm, i.d., S-5 μm, YMC Co. Ltd., Japan), the chromatogram is viewed at 254 nm. 0.3% TFA/MeCN:0.3% TFA/water, 95:5 (v/v) was used as the mobile phase in an isocratic mode at a flow rate of 3.0 mL/min, peaks at 5.4 min were pooled and lyophilized. The desired compound, S-NMN (60.8mg, 29%) was obtained as colorless oil. To improve the purity of the sample, two or three rounds of purification were necessary. 1H NMR (300 MHz, D_2O) *delta* 9.32 (s, 1H), 9.20 (d, 1H, *J* = 6.0Hz), 8.83 (d, 1H, *J* = 7.5 Hz), 8.17 (d, 1H, *J* = 6.9 Hz), 6.09 (d, 1H, *J* =5.1 Hz), 4.54 (brs, 1H), 4.67 – 4.53 (m, 1H), 4.33 – 4.32 (m, 1H), 4.24 – 4.07 (m, 2H) ppm. HR ESI-MS (m/z): calculated for $C_{11}H_{16}N_2O_7PS^+$: 351.0410, found: 351.0414.

Nucleotides extraction and cycling assay

Cells were pelleted and lysed with 0.6 M perchloric acid. After centrifugation, the cADPR or NAD amount in the supernatant was measured by a cycling assay described previously(Graeff & Lee, 2002), and the pellets were re-dissolved in 1 M NaOH and quantified by Bradford assay (Quick Start™ Bradford Kit, BIO-RAD). Results were presented as picomole cADPR per milligram of total proteins, or picomole NAD per milligram of total proteins.

The NMN contents were quantified by a fluorometric enzyme-coupled assay (Zamporlini, Ruggieri et al., 2014). Briefly, NMN was firstly adenylylated to NAD by the recombinant NMNAT1(Schweiger, Hennig et al., 2001). The resulted NAD was quantified by a cycling assay. Basically, the assay solution containing 2.5 mM $MgCl_2$, 0.33 mM ATP, 0.5 mg/mL BSA, 200 ng/mL NMNAT1, 2% ethanol, 0.1ug/mL alcohol dehydrogenase (ADH), 10 μM resazurin, 55 μg/mL diaphorase, 10 μM FMN, and 100 mM Na_2HPO_3 buffer, pH 7.0 was added to each sample and the kinetics of fluorescence changes were monitored as previously described.

Ca^{2+} release in sea urchin homogenate

Homogenates of sea urchin eggs were prepared and used as a bioassay as described previously(Lee & Aarhus, 1991). Briefly, frozen egg homogenates (25%) of sea urchin (*Strongylocentrotus purpuratus*) were thawed in a GluIM medium containing 250 mM N-methylglucamine, 250 mM K-gluconate, 20 mM Hepes, 1 mM $MgCl_2$, 2 units/mL creatine kinase, 8 mM phosphocreatine, 0.5 mM ATP and 3 μM fluo-3, pH 7.2. The homogenates were diluted to 1.25 % with the homogenate at 17 °C. Ca^{2+} release was measured in an Infinite M200 PRO microplate reader (Tecan) (ex/em: 490/535 nm) by adding 2 μL of purified compound from the extract or cADPR standards to 200 μL of homogenates. On the linear portion of the standard curve, cADPR concentration in the purified sample was calculated.

Immunoprecipitation and Western blots

Whole-cell lysates were prepared in ice-cold lysis buffer (50 mM Tris (pH 8.0), 150 mM NaCl, 1 mM EDTA, 0.5% TritonX-100). To extract SARM1-Flag, HEK-293 cells stably expressing SARM1-Flag were treated with KHM buffer (110 mM potassium acetate, 20 mM Hepes (pH 7.4), 2 mM $MgCl_2$) containing 0.1 mM digitonin and protease inhibitor cocktail (Roche) for 5 min. After centrifugation, the supernatants were collected and subjected to εNAD assay, or further IP(Bonifacino & Dell'Angelica, 2001) by Anti-FLAG M2 Magnetic Beads (Sigma) and eluted by 3x Flag peptide for the enzymatic assays and other applications.

SARM1-dN-HEK-293T cells were harvested and lysed by 0.1 mM digitonin in KHM buffer with protease inhibitor cocktail for 5 min. For single step affinity purification, the supernatants were incubated with BC2TNb-NHS beads for 4h. The beads were then washed three times with KHM buffer and resuspended for measurement of the multiple enzymatic activities by HPLC.

Western blots were done with different antibodies indicated in the main text and the signals were developed by ECL (Abvansta), detected and quantified by a Chemidoc MP system and ImageLab software (BIO-RAD).

Measurement of NADase activity by the fluorescence assay

Briefly, 25 μg of lysate contains SARM1-Flag, prepared as described above, were applied to 100 μL of 50 μM εNAD in KHM buffer with or without different compounds. Kinetic fluorescence reading (ex/em: 300 nm/410 nm) was immediately started after adding εNAD in an Infinite M200 PRO microplate reader (Tecan), maintained around 1.5 hours. The slopes of fluorescence kinetics (RFU/min) were calculated and used to quantify the activities of SARM1.

Measurement of enzymatic activities by HPLC

Around 700 nM purified SARM1-dN (with or without pre-treatment 100 μM NMN at room temperature) or 1 nM reCD38 (as a positive control), was incubated with 100 μM NAD or 100 μM cADPR, 100 μg/mL BSA in KHM (pH 7.4) for different time periods. The base-exchange reaction was assayed using 100 μM NADP and 2.5 mM nicotinic acid in 15 mM HAc (pH 4.5). The total volume of the reaction mixture was 110 μL, and the

reaction was stopped by the addition of 0.2% SDS. The protein was removed by filtration using Immobilon-P plates (Millipore). 90 µL of the mixture was injected to an HPLC system with an AG MP-1 column (Bio-Rad) with a gradient of trifluoroacetic acid as described previously (Munshi, Aarhus et al., 2000). The identities of the peaks were determined by the retention time on the column with the pure nicotinamide, NAD, NADP, cADPR, ADP-ribose, NAADP, NMN as the references.

Immunofluorescence microscopy and co-localization analysis

HEK-293 cells were grown on poly-L-lysine coated coverslip (ULAB Supply, Nanjing, China). Forty-eight hours after transfection, the cells were fixed with 4% PFA, permeabilized with 0.5% Triton X-100, blocked by 1% BSA and stained with rabbit anti-SARM1 (Absea Biotechnology Ltd, China) and mouse anti-Tom20 (Santa Cruz Biotechnology) for 1.5h, followed by incubation of Alexa Fluor-conjugated donkey anti-rabbit or anti-mouse secondary antibody. Cells were then stained with DAPI for 5 min, mounted. Images were acquired by Nikon A1 Confocal Laser microscope. NIS-Elements AR (Advanced Research) software was used to do the colocalization analysis of the confocal signals.

Protein-fragment complementation assay (PCA)

The cells (5×10^4 cells per well) were plated in 96-well flat-bottomed white microtiter plates and the PCA was performed accordingly(Liu et al., 2017, Remy & Michnick, 2006). Native coelenterazine (Nanolight Technology) working solution of 20 µM was auto-injected to the wells with cells. Luminescence signals were recorded on an Infinite 200 PRO plate-reader (TECAN).

Cell death analysis

Wildtype HEK-293 cells or SARM1-overexpressing cells were treated with 100 µM CZ-48 for the indicated time. The cell morphology was recorded under a Nikon microscope equipped with a NAMC module. The cells were then resuspended in ice cold PBS containing 5 µl of FITC-Annexin V and 5 µL (1 µg/mL) of propidium iodide (PI) each sample, and incubated for 20 min at room temperature (25℃) in the dark. Stained cells were analyzed by flow cytometry using FL1 (green, Annexin V-FITC) and FL3 (red, PI) channels.

Measurement of ATP levels

The cellular nucleotides were extracted by 0.6 M perchloric acid and neutralized as those in cADPR and NMN quantification. The extracts and the ATP standards, were incubated with 250 µM luciferin, 1 mM EDTA, 250 µM Coenzyme A, 1 mg/mL BSA, 64 mM dithiothreitol, 0.1 µg/mL Nb1053-Fluc2(Li, Li et al., 2018), 10 mM $MgCl_2$ and 200 mM Tris-HCl (pH 7.8), and luminescence were recorded in an Infinite 200 PRO plate-reader (TECAN). The concentrations of ATP in the samples were determined by the luminescence and the standard curve. All assays were performed at least in triplicate.

Analysis of ROS increase

To determine the concentration of the mitochondrial superoxide, the cells were incubated with 5 μM MitoSOX Red for 15 min at 37℃. The fluorescence signals (ex/em: 488 nm/580nm) were analyzed by CytoFLEX (Beckman Coulter).

Mitochondrial membrane depolarization

To determine the mitochondrial membrane potential, the cells were incubated with 40 nM of 3,3' dihexyloxacarbocyanine iodide (DiOC6(3)) for 30 min at 37 ℃. The fluorescence signals (ex/em: 482nm/504nm) were analyzed by CytoFLEX (Beckman Coulter).

RNA extraction and qPCR

Total RNAs were extracted using the E.Z.N.A.® Total RNA Kit I (Omega Bio-tek, Georgia) and 1 μg RNAs were reversely transcribed into cDNA using the TransScript First-Strand cDNA Synthesis SuperMix (TransGen Biotech, Beijing). Quantification PCR was performed with TransStart Green qPCR SuperMix kit (TransGen Biotech, Beijing) on a Bio-Rad CFX machine (Bio-Rad Laboratories, USA) according to the manufacturer's instructions. The relative expression levels were calculated by normalizing with *actin*, a housekeeping gene, with the ΔΔCt method. Primers used: *Sarm1* (5'-CTGGACAAGTGCATGCAAGA-3'; 5'- GGTGGCCTCCTGGTATTCGT-3') and *Actin* (5'- CCTGGCACCCAGCACAAT-3'; 5'- GGGCCGGACTCGTCATACT-3').

Data analysis

All experiments contained at least three biological replicates. Data shown in each figure are all means ± SD. The unpaired Student's *t*-test was used to determine statistical significance of differences between means (*P<0.05, **P<0.01, ***P<0.001, ****P<0.0001). GraphPad Prism 7 was used for data analysis.

Supplementary Figures

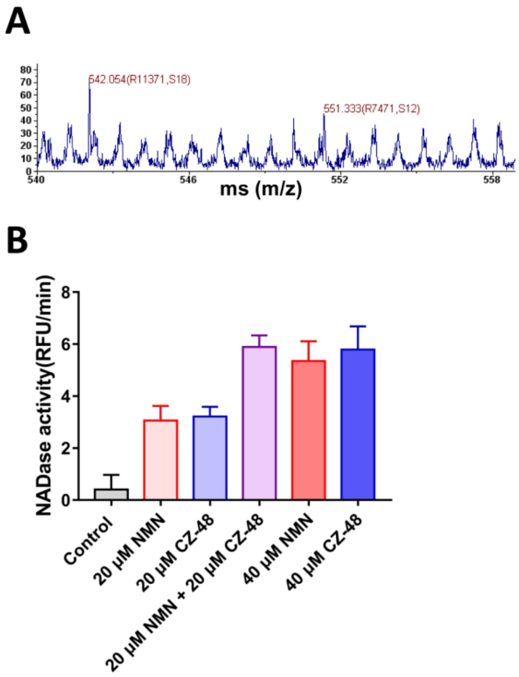

Figure S1. Related to Figure 1 and 3C.

(A) characterization of the CZ-48 induced compound

The fractions 4, 5 and 6 collected from HPLC (Figure 1B, green box) were merged (named as "Peak 13") and analyzed by MOLDI-TOF. The molecular weight of the target compound in Peak 13 is 542.054 Da.

Methods: The extract was applied to HPLC equipped with a column filled with AG MP-1 resin (Bio-rad). The nucleotides were eluted with a gradient of trifluoroacetic acid as described previously(Munshi et al., 2000) and sampled at a constant interval. The collected samples were applied to cycling assay. The fractions from HPLC with the high values in cycling assay were merged, lyophilized, re-dissolved in water and applied to a MOLDI-TOF instrument (Center for Genomic Sciences, HKU) for determination of molecular mass.

(B) Supplementary data for Figure 3C to test whether the effects of CZ-48 and NMN were additive. Lysates of SARM1-Flag cells were incubated with the indicated compounds and the NADase activities were measured as Figure 3C. Control: adding vehicle.

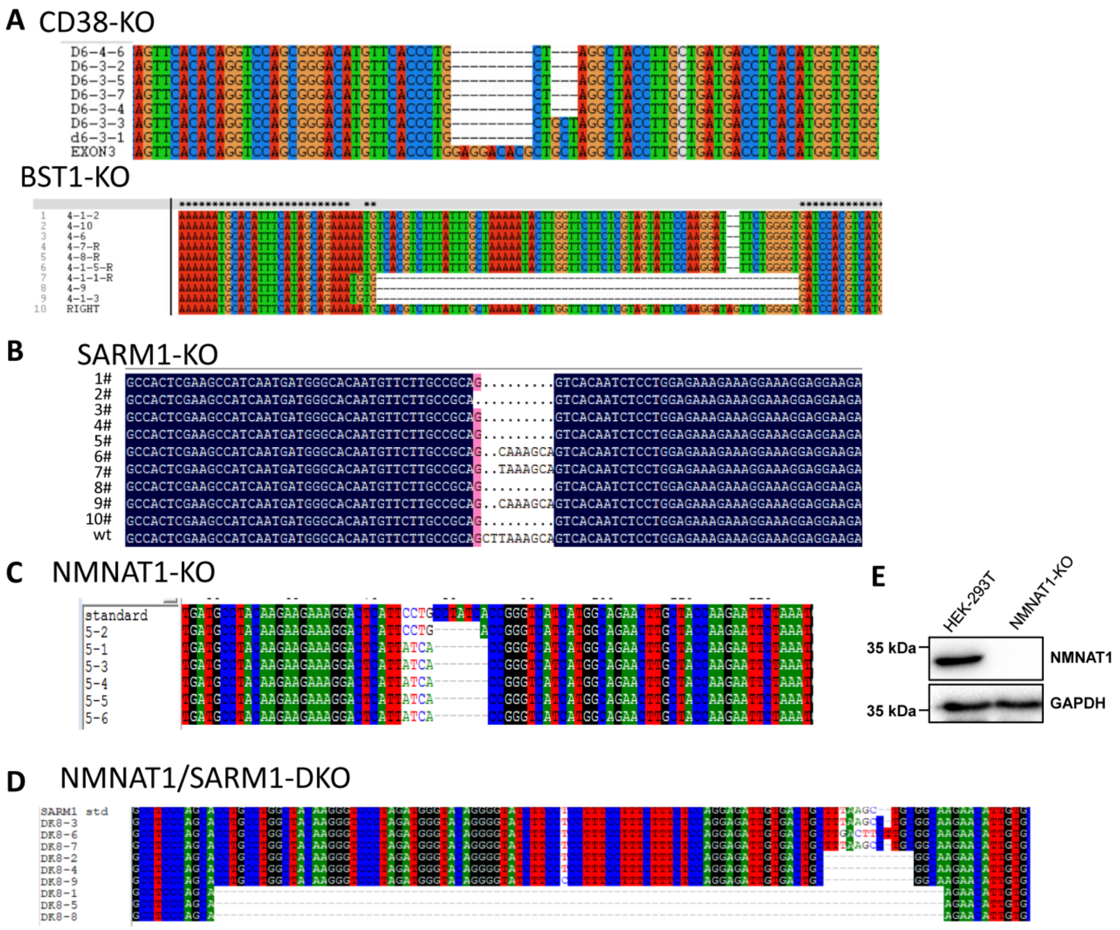

Figure S2. Related to Figure 2A-B and 3E-G. Validation of gene knockout cell lines.

(A) DNA sequencing of CD38/BST-1 double knockout HEK-293T cells.

(B) DNA sequencing of SARM1 knockout HEK-293T cells.

(C) DNA sequencing of NMNAT1 knockout HEK-293T cells.

(D) DNA sequencing of NMNAT1/SARM1 double knockout HEK-293T cells, constructed on the basis of NMNAT1-KO cells shown in (C).

There are at least two different types of deletions in each gene and no wildtype sequence was founded, suggesting both alleles of genes in two chromosomes were knocked out.

(E) The expression of NMNAT1 was analyzed by Western blots in wildtype and NMNAT1-knockout HEK-293T cells.

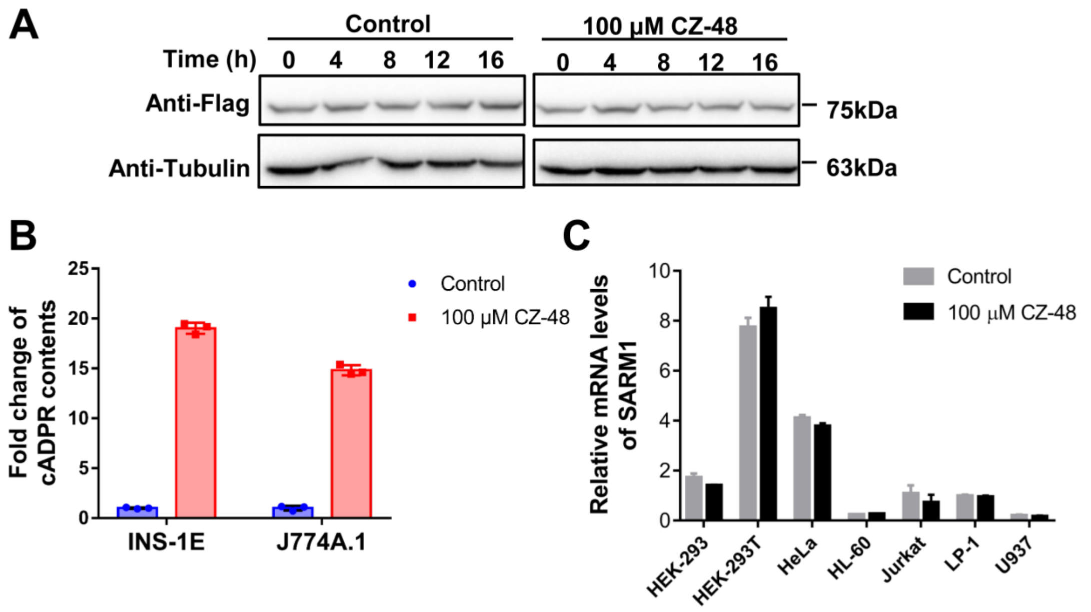

Figure S3. Related to Figure 2C, 2E and 2F.

(A) The supplementary data for Figure 2C. HEK-293 cells, carrying an inducible expression cassette of Flag-tagged SARM1 were treated with 100 μM CZ-48 or vehicles and the expression levels of SARM1-Flag were analyzed by Western blots.

(B) The cell lines were treated with 100 μM CZ-48 for 24 h. The cADPR levels were measured by cycling assay.

(C) The supplementary data for Figure 2F. The mRNA levels of SARM1 in the cell lines were quantified by qRT-PCR before and after treatment with 100 μM CZ-48 for 24 h.

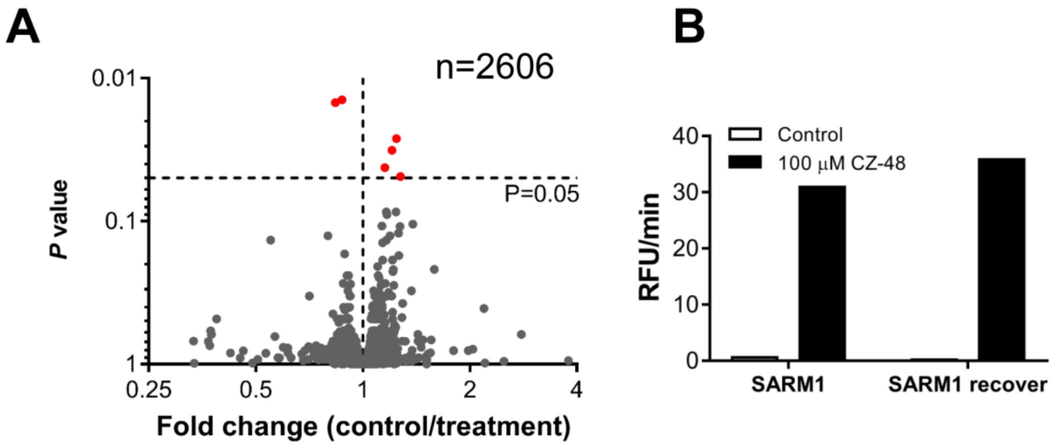

Figure S4. Related to Figure 3.

(A) Volcano plots of metabolites in response to cells treated with CZ-48. Red dots represent metabolites (663.1089@3.9329998, Fluorofelbamate, 4-(Trimethylammonio)but-2-enoate Esi+6.5569997, 664.1116@3.9539998, Ectoine, 495.1544@6.5609994 and 77.0265@1.666) significantly changed between control and treated conditions (P < 0.05), although the fold changes were all less than 2. Data are mean of six biological replicates.

Methods: SARM1-KO HEK-293T cells were treated with 100 μM CZ-48 for 24 h, untreated as controls, lysed by 0.6 M perchloric acid (PCA) and the metabolites were extracted by chloroform:tri-n-octylamine (3:1), as described previously(Graeff & Lee, 2002). The aqueous samples containing metabolites were analyzed on a 1290 infinity II UPLC coupled with 6545 Q-TOF system with positive and negative ESI modes and a HILIC column (Agilent Technologies, Inc.). Overall, we processed and analyzed a total of 12 samples, including 2 groups (control and treatment) and 6 replicates for each group. Non-targeted metabolite profiling, peak identification, and curation was performed by MassHunter Software (Agilent Tech. USA). Briefly, after initial processing of the LC/MS data by MassHunter Qualitative Analysis software, the Molecular Feature Extractor (MFE) combined with Find by Formula (FBF) tool was used to mine and recursively find compounds by using the presence of isotope ions and other adduct ions (other than M plus H in Pos. mode and M minus H in Neg. mode). And this procedure allows us to eliminate noise and filter out those compounds with a minimum abundance (peaks with counts ＜5000) and frequency (peak frequency of occurrences ＜50% data within condition). Profiling of the MS data was accomplished using the Agilent Mass Profiler Professional (MPP) software, version 14.9 (Agilent Technologies, Inc.) to align mass and retention time data across the samples within the sets, and to define the parameters for the various profiling tests in the project. An output file was obtained after data processing that included detected m/z and relative intensity in the different samples. The volcano plot was generated using GraphPad Prism 7.

(B) The binding of CZ-48 to SARM1 was non-covalent. With or without 100 μM CZ-48 pre-treatment, the lysate containing SARM1-Flag was washed with KHM buffer by Centricon filters. The activities of the recovered proteins were assayed by εNAD in absence or presence of 100 μM CZ-48.

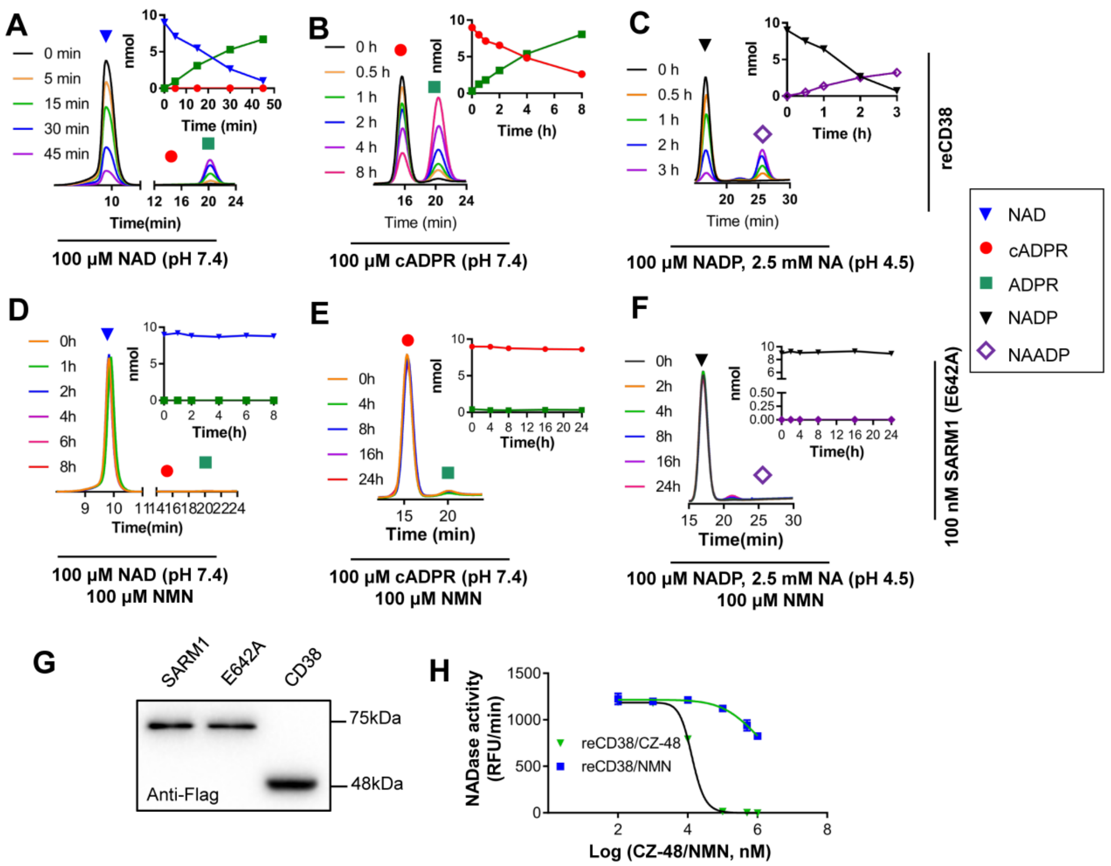

Figure S5. Related to Figure 4 and 3C.

(A-C) The activities of reCD38.

(A) The same NAD hydrolysis reaction and analysis as Figure 4A, B except the enzyme was 1 nM reCD38.

(B) The same cADPR hydrolysis reaction and analysis as Figure 4C, D except the enzyme was 10 nM reCD38.

(C) The same NAADP synthesis reaction and analysis as Figure 4E, F except the enzyme was 1 nM reCD38.

(D-G) The SARM1(E642A)-Flag was immunoprecipitated by anti-Flag Magnetic beads and eluted by 3x Flag peptide. The purified SARM1(E642A)-Flag was quantified by Western blots (G). Around 100 nM of SARM1(E642A)-Flag, in presence of 100 µM NMN, was used in three reactions.

(D) The activities of NAD hydrolase and ADP-ribosyl cyclase. The dead enzyme was incubated with 100 µM NAD in KHM (pH 7.4) for different time periods and the products were analyzed by HPLC. Insets: quantification of the products. Blue triangles, NAD; Red circles, cADPR; Green squares, ADP-ribose.

(E) cADPR hydrolase activity. Similar reactions were set and analyzed as (D), except the substrate was replaced by same amount of cADPR. Insets: Red, cADPR; Green, ADP-ribose.

(F) Base-exchange reaction. Similar reactions were set and analyzed as (D), except the substrate was replaced by same amount of NADP and 2.5 mM NA in 15 mM acetate

buffer (pH 4.5). Insets: black: NADP; purple: NAADP.

(H) Similar experiments as Figure 3C. reCD38 were prepared as previously described(Munshi, Fryxell et al., 1997). The NADase activities of reCD38 (200 ng/mL) were assayed by εNAD co-incubated with different doses of NMN or CZ-48.

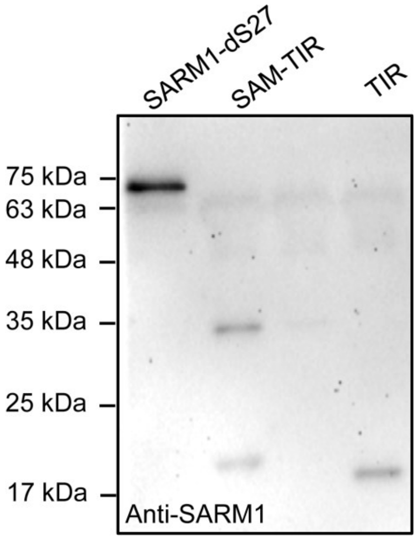

Figure S6. Related to Figure 5F. Western blots of the proteins.

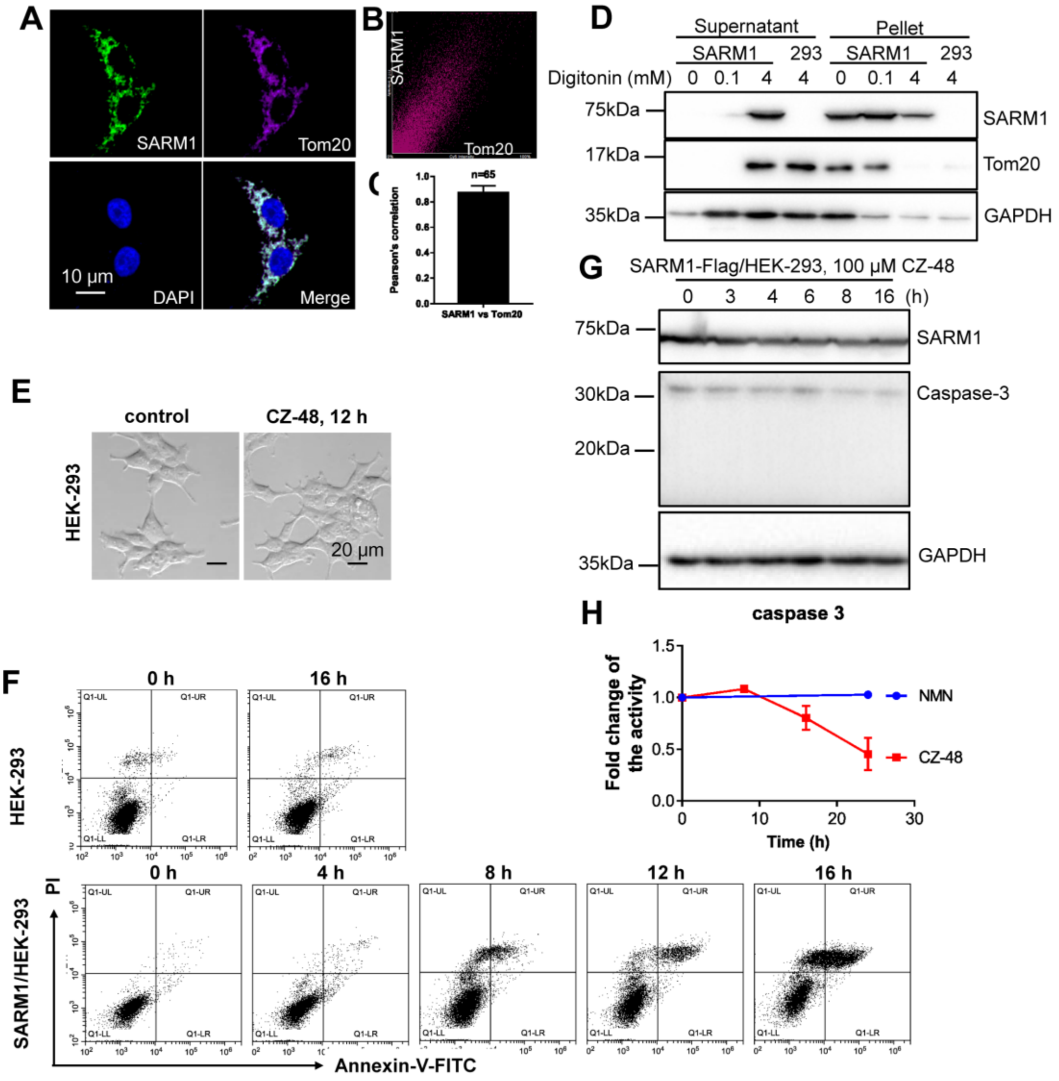

Figure S7. Related to Figure 6.

(A) SARM1-overexpressing HEK-293 cells were co-immunostained with anti-SARM1 and anti-Tom20 and imaged by confocal microscopy. Tom20 is a mitochondrial out-membrane protein, serving as a mitochondrial marker. DAPI is for nuclear counterstains. Scale bars: 10 μm.

(B) Co-localization between SARM1 and Tom20 were analyzed with NIS-Elements AR software. Scatter plot pixels correspond to the images shown in (A). A complete colocalization results in a pixel distribution along a straight line whose slope will depend on the fluorescence ratio between the two channels.

(C) Pearson's correlation coefficient (PC) was analyzed with NIS-Elements AR software. Values shown was Mean ± SEM of 65 cells.

(D) SARM1-overexpressing cells were treated with lysate buffers containing 0.1 or 4 mM of digitonin and the protein amounts of SARM1 in the supernatants or pellets were analyzed by Western blots, together with the mitochondrial out membrane protein, Tom20 and cytosolic protein, GAPDH as the controls.

(E) The supplementary data for Figure 6A. CZ-48 treatment did not induce the cell morphological changes in HEK-293 cells. Scale bars: 20 μm.

(F) supplementary diagram of flow cytometry for Figure 6C.

(G) CZ-48 treatment did not induce activation of caspase-3. HEK-293 cells overexpressing SARM1-Flag were treated with 100 μM CZ-48 and the cleavage of caspase-3 was not observed in Western blot. Anti-caspase 3 (cat no. 66470) was from Proteintech, Ltd.

(H) The activities of caspase-3 in the cell lysate were measured with a caspase fluorometric assay kit (cat.no. K105, BioVison). HEK-293 cells overexpressing SARM1-Flag were treated with 100 μM CZ-48 or NMN for the indicated time. The lysates were incubated with 50 μM DEVD-AFC substrate at 37℃ for 1.5h and the fluorescence signals (ex/em: 400nm/505nm) were measured by the plate-reader (TECAN). The activities were normalized by those of the samples without treatment. The results showed that caspase-3 was not activated, but significantly inhibited after CZ-48 treatment.

Reference

Bonifacino JS, Dell'Angelica EC (2001) Immunoprecipitation. Current protocols in cell biology Chapter 7: Unit 7 2

Chen Y, Stevens B, Chang J, Milbrandt J, Barres BA, Hell JW (2008) NS21: re-defined and modified supplement B27 for neuronal cultures. Journal of neuroscience methods 171: 239-47

Clapper DL, Walseth TF, Dargie PJ, Lee HC (1987) Pyridine nucleotide metabolites stimulate calcium release from sea urchin egg microsomes desensitized to inositol trisphosphate. The Journal of biological chemistry 262: 9561-8

Graeff R, Lee HC (2002) A novel cycling assay for cellular cADP-ribose with nanomolar sensitivity. The Biochemical journal 361: 379-84

Kwong AK, Chen Z, Zhang H, Leung FP, Lam CM, Ting KY, Zhang L, Hao Q, Zhang LH, Lee HC (2012) Catalysis-based inhibitors of the calcium signaling function of CD38. Biochemistry 51: 555-64

Lee HC, Aarhus R (1991) ADP-ribosyl cyclase: an enzyme that cyclizes NAD+ into a calcium-mobilizing metabolite. Cell regulation 2: 203-9

Li T, Li SL, Fang C, Hou YN, Zhang Q, Du X, Lee HC, Zhao YJ (2018) Nanobody-based dual epitopes protein identification (DepID) assay for measuring soluble CD38 in plasma of multiple myeloma patients. Analytica chimica acta 1029: 65-71

Liu J, Zhao YJ, Li WH, Hou YN, Li T, Zhao ZY, Fang C, Li SL, Lee HC (2017) Cytosolic interaction of type III human CD38 with CIB1 modulates cellular cyclic ADP-ribose levels. Proceedings of the National Academy of Sciences of the United States of America

Munshi C, Aarhus R, Graeff R, Walseth TF, Levitt D, Lee HC (2000) Identification of the enzymatic active site of CD38 by site-directed mutagenesis. The Journal of biological chemistry 275: 21566-71

Munshi CB, Fryxell KB, Lee HC, Branton WD (1997) Large-scale production of human CD38 in yeast by fermentation. Methods Enzymol 280: 318-30

Remy I, Michnick SW (2006) A highly sensitive protein-protein interaction assay based on Gaussia luciferase. Nat Methods 3: 977-9

Schweiger M, Hennig K, Lerner F, Niere M, Hirsch-Kauffmann M, Specht T, Weise C, Oei SL, Ziegler M (2001) Characterization of recombinant human nicotinamide mononucleotide adenylyl transferase (NMNAT), a nuclear enzyme essential for NAD synthesis. FEBS letters 492: 95-100

Sleigh JN, Weir GA, Schiavo G (2016) A simple, step-by-step dissection protocol for the rapid isolation of mouse dorsal root ganglia. BMC research notes 9: 82

Zamporlini F, Ruggieri S, Mazzola F, Amici A, Orsomando G, Raffaelli N (2014) Novel assay for simultaneous measurement of pyridine mononucleotides synthesizing activities allows dissection of the NAD(+) biosynthetic machinery in mammalian cells. FEBS J 281: 5104-19
